# Intermittent Fasting: Myths, Fakes and Truth on This Dietary Regimen Approach

**DOI:** 10.3390/foods13131960

**Published:** 2024-06-21

**Authors:** Simone Brogi, Rita Tabanelli, Sara Puca, Vincenzo Calderone

**Affiliations:** 1 Department of Pharmacy, University of Pisa, Via Bonanno 6, 56126 Pisa, Italy; ritatabanelli@gmail.com (R.T.); saravictorlive@hotmail.it (S.P.); vincenzo.calderone@unipi.it (V.C.); 2 Bioinformatics Research Center, School of Pharmacy and Pharmaceutical Sciences, Isfahan University of Medical Sciences, Isfahan 81746-73461, Iran

**Keywords:** intermittent fasting (IF), alternate day fasting (ADF), time-restricted feeding (TRF), obesity, dietary regimen

## Abstract

Intermittent fasting (IF) has been indicated as a valuable alternative to the classical caloric restriction dietary regimen for lowering body weight and preventing obesity-related complications, such as metabolic syndrome and type II diabetes. However, is it effective? In this review article, we analyzed over 50 clinical studies in which IF, conducted by alternate day fasting (ADF) or time-restricted feeding (TRF), was compared with the caloric restriction approach. We evaluated the different roles of IF in treating and preventing human disorders such as metabolic syndrome, type II diabetes, and some types of cancer, as well as the usefulness of IF in reducing body weight and cardiovascular risk factors such as hypertension. Furthermore, we explored the cellular pathways targeted by IF to exert their beneficial effects by activating effector proteins that modulate cell functions and resistance to oxidative stress. In contrast, we investigated concerns regarding human health related to the adoption of IF dietary regimens, highlighting the profound debate surrounding weight loss regimens. We examined and compared several clinical trials to formulate an updated concept regarding IF and its therapeutic potential.

## 1. Introduction

**Figure 1:**
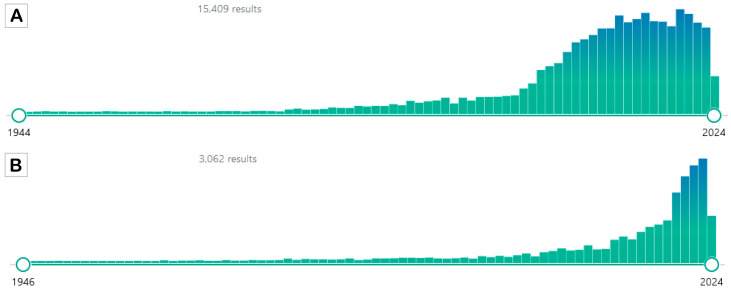
PubMed results for the two search attempts. Panel (**A**) shows the results of the search using the terms “caloric restriction” OR “calorie restriction”. Panel (**B**) shows the results of the search using the terms “intermittent fasting” OR “alternate day fasting”. The search was performed on 23 May 2024 (source PubMed https://pubmed.ncbi.nlm.nih.gov/; accessed on 23 May 2024).

Among the current dietary regimens leading to weight loss, reducing risk factors for several disorders, including hypertension, dyslipidemia, obesity, inflammation, insulin resistance, and more in general metabolic syndrome (MetS), and improving health benefits over aging-dependent diseases, intermittent fasting (IF) has gained significant attention as a practical nutritional strategy (Figure 1) [1,2,3,4]. In fact, IF does not adhere to a rigid eating schedule (no calorie counting), and people who choose to follow it universally do so because it allows them to be flexible with the items they eat. Furthermore, IF is easy to implement in existing dietary regimens, and it is also a reasonably effective, low-maintenance approach for improving health. Therefore, IF could offer some advantages over classical calorie restriction (CR), although the main goal of both CR and IF is to limit the intake of energy. The main distinction was that although IF consumed little or no food during fasting, CR continued to eat regularly [5]. Currently, CR is considered the typical dietary method for losing or maintaining weight; however, it has been proven to be difficult to sustain for many people, with a high risk of late weight rebound when used for an extended period. Thus, some improvements in this scenario have been achieved by combining intermittent CR and temporal management, replacing the basic long-term CR regimen [6]. In fact, it has been established that adherence to CR, in which a reduction of 20–40% of caloric intake occurs, sharply declines over a long period of time, and many people acquire noticeable weight by the year’s end [7,8]. In contrast, IF has been hypothesized to be superior in terms of patients’ compliance [9,10,11], although other studies revealed that IF’s high dropout rate—which was 38% for IF compared with CR—limited its ability to maintain compliance over the long run [12,13]. In the mid-term, the absence of a need to control calories during the eating day could be one of the reasons for better patient compliance. In general, considering the purpose of the two approaches, the effects of CR and IF substantially overlap across numerous aspects [14]. However, several studies have suggested that restricting protein intake in the diet can increase lifespan and suppress the incidence of age-related diseases. In particular, amino acid restriction approaches, such as methionine restriction (MR), are believed to exert benefits on lifespan extension and metabolic health [15,16,17,18]. In fact, low-methionine diets appear to improve overall metabolic health by decreasing body weight, body mass index (BMI), and serum leptin concentrations and increasing plasma concentrations of the ketone bodies β-hydroxybutyrate (BHB) and acetoacetate in overweight and obese adults [16]. MR also resulted in decreased uric acid, blood urea nitrogen (BUN), and 8-isoprostane levels and increased hepatic fibroblast growth factor 21 (FGF-21) levels in healthy adults [15]. In contrast to CR and IF, the effects of MR on energy balance, adiposity, and insulin signaling do not require food restriction [18]. However, several studies have reported a lack of adherence and a high dropout rate, likely due to the synthetic nature of the diet [19]. In fact, dietary methionine is contained in animal sources of protein—an important dietary source of micronutrients—which is essential to consider in order to prevent deficiencies and malnutrition [20]. Because many vegan diets naturally contain low levels of methionine, plant-based foods have become the main strategy for increasing palatability and adherence to the MR regimen [15,16,21]. Notably, methionine is an essential amino acid required for normal developmental processes, and its intake substantially decreases after early adulthood [21]. Regarding IF, in a specific context, it could provide superior effectiveness than CR; thus, in this article, a fine analysis was conducted to better understand situations for which IF could be preferred to CR and vice versa to help the scientific community, patients, and individuals who need specific nutritional interventions like these.

According to these clues, IF has been investigated in clinical trials applying different IF regimens because different IF approaches have been developed and are discussed in the next paragraph. However, from the first application of the IF dietary regimen, the still controversial question is “in improving health benefits, including weight loss, is IF superior to CR?” In this article, we attempt to offer an outlook on IF practice by discussing the advantages and discordances regarding the application of the mentioned nutritional interventions. We analyzed several representative clinical studies for different purposes with different endpoints to provide evidence to answer the aforementioned question. Accordingly, being a narrative review, we did not apply selective or specific inclusion criteria, but we have considered several clinical studies, preferring randomized controlled trials that compared IF and CR (search performed in PubMed, Google(Scholar), ResearchGate, and Scopus, inserting several combinations of keywords, including “clinical study”, “clinical trial”, “intermittent fasting”, “alternate day fasting”, “intermittent fasting” AND “caloric restriction”/“calorie restriction”, “time restricting eating/feeding/fasting”, “weight”, “metabolic syndrome”, “obesity”, “obese”, “overweight”, “cardiovascular”, “neurological”, neurodegeneration”, “neurodegenerative”, “cancer”, “disorder(s)”, “disease(s)”, “history”, “political”, “religion”, “religious”, “cellular pathway”, “advantages”, “disadvantages”, “pros” AND “cons”), that were, from our personal point of view, relevant (i.e., the first studies in the considered fields, studies with controversial results, etc.) in evaluating the use of IF, starting a critical discussion, as a significant nutritional intervention in different types of disorders with a special focus on weight loss.

Before entering into the critical analysis of the clinical studies analyzed here, we would like to start this manuscript with a brief mention of the origin of fasting and its relative religious and social implications, which opened the way to the formulation of nutritional interventions in which fasting is crucial, which are still present in some populations and ethnicities.

### 1.1. Fasting: A Historical and Social View

For medical, spiritual, or political purposes, fasting is generally understood as an intentional period of time during which one abstains from eating [22]. This practice has been known since ancient times, and several philosophers and physicians, such as Socrates, Hippocrates, Galen, Aristoteles, Paracelsus, Plato, and several religious communities, documented fasting in ancient writings as having physiological or spiritual benefits [23,24,25]. For example, Plato, who wrote: ”I fast for greater physical and mental efficiency,” based his idea of food consumption on moderation, indicating that excess feeding should be deprecated because of this behavior, which leads to the development of various diseases. Remarkably, for a healthy body, he indicated aliments that should be assumed with strong frequency (fish, legumes, milk, cereals, honey, and fruits), whereas with significant moderation, confectionery, meat, and wine could be eaten. Plato’s nutritional indications shared several features with the Mediterranean diet [23]. Again, “Fasting is the greatest remedy—the physician within,” is attributed to Paracelsus, one of the three founding fathers of Western medicine. In agreement with the recommendation provided by Hippocrates, a Greek physician who suggested drink and/or food abstinence for patients showing evident symptoms of disorders wrote “if you eat when you are sick, it will make you sick” [26]. Historically, religious fasting was a common divinatory practice that involved pursuing certain types of food abstinence for spiritual purposes [20]. In fact, Christianism acknowledges 40 days of fasting in the desert as preparation for divine revelations, as described in the Old Testament. Furthermore, local Christian churches gradually adopted conventions of fasting, in part to supplant earlier pagan and Jewish fasting practices [27]. Moreover, one of the earliest documented instances of severe starvation in history was St. Catherine of Siena during the Christian era. Her diet consisted solely of vegetables and water, which she self-imposed as a restrictive routine. This was among the earliest types of holy fasting, clearly inspired by a strong sense of religious conviction. Known by the name inedia prodigiosa, this disorder is categorized as anorexia mirabilis [28]. Finally, the monastic practice of fasting had great prosperity in the fourth and fifth centuries, with asceticism—driven by a sense of penance and self-humiliation as a monk sought communion with his God—serving as the primary motivation [29]. In the medieval period, women frequently emulated St. Catherine, who professed to only eat the Eucharist in order to demonstrate her purity, penitence, devotion, and strength of spirit. The issue of repeated holy fasting was quickly recognized by the clergy, who responded with detailed rules emphasizing good deeds over fasting for beatification. Anorexia mirabilis appeared to vanish during the Renaissance, only to resurface later as a form of protest, heretical, socially harmful, and occasionally thought to have Satanic roots [30]. Again, in Judaism, a lot of nutritional rules have been proposed. Accordingly, believers observe diverse fast days within a year, principally on days of penitence (i.e., Yom Kippur, the Day of Atonement on which religious fasting is observed on the first day of the seventh month of the Hebrew calendar. It is anticipated that giving up eating pleasure will enhance one’s capacity to concentrate on repentance. The Yom Kippur fast persists for 25 h, starting before nightfall on the evening before the holiday and ending after sunset on the actual day of Yom Kippur) [31,32].

A more structured religious fasting approach is Ramadan, a pillar of Islam, through which fasting believers seek to achieve soul purity. According to this religious practice, millions of Muslims are asked to abstain from food and liquids during the fasting month of Ramadan, which spans 28–30 days, from sunrise (Sahur) to sunset (Iftar) each year. Interestingly, because both fasts include feast and fast periods, Ramadan fasting and a modified IF approach, namely alternate day fasting (ADF, that is detailed in the next paragraph), are comparable. During Ramadan, the feast and fast periods last an average of 12 h each. Drinking liquids is prohibited during Ramadan fast periods, but it is allowed at all times under any IF nutritional regimen, which represents a marked distinction between the two types of fasting [27,33]. Traditionally, Muslims who fast throughout Ramadan have one major meal after sunset and one smaller meal before dawn. However, some Muslims eat one more meal before going to bed [34]. During Ramadan, Muslims eat a wider range of meals than they do during the year. Additionally, during Ramadan, sweet foods and beverages are consumed more frequently [35]. However, there are significant differences in managing this religious nutritional practice on the basis of different geographical areas, fasting duration, methodological approaches, medications, dietary habits, seasonal changes, daylight exposure, cultural norms, and physical activity [27]. Probably for these reasons, several empirical investigations of this feeding approach did not reach convergent results with respect to healthy benefits, including nutrient intake, improvements in BMI and body weight [36,37], blood pressure [38,39], total cholesterol, low-density lipoprotein cholesterol (LDL-C), and high-density lipoprotein cholesterol (HDL-C). Furthermore, there are conflicting results regarding whether fasting during Ramadan lowers or raises the LDL-C/HDL-C ratio [39,40,41]. Lastly, it seems that during Ramadan, the ratio of total cholesterol to HDL-C decreases [27]. Blood glucose levels and the total lipid profile are also found to have similar inconsistencies [42,43].

Finally, fasting has also been identified as a usual instrument for political protests in the contemporary period. The main actor in fasting for political purposes was Mahātmā Gandhi. Throughout his life, he fasted at least 14 times, and three times the abstinence from food was longer than 21 days. He wrote: “When you fast, the Light will illuminate you and spread on earth” [44]. During the English-Irish social and political conflict, several episodes of fasting by Irish activists have been recorded. In 1920, Terence Mac-Swiney, a politician who was elected mayor of Cork after his detention, fasted for 74 days until his death. On the same day, another Irish activist, Joseph Murphy, who had protested for 76 days in a hunger strike, passed away [26]. Later, several components of the Irish Republican Army fasted to protest the inhumane conditions of the Maze prison in Belfast. In this context, it is emblematic of the death of a member of the mentioned group, Bobby Sands, after 66 days of fasting [45]. Cuban dissidents during the era of Fidel Castro, such as Pedro Luis Boitel (1961), Guillermo Fariñas (2006), Jorge Luis García Pérez (2009), and Orlando Zapata (2010), protested by hunger strike to bring to attention the conditions of dissidents, the political action of the government, and the censorship of information, including the Internet. Unfortunately, some of these activists died because of starvation [46,47].

In our country, fasting for political reasons has been described several times. In particular, several protests for civil rights in the last century interested Italy. The Italian politician Marco Pannella, the historic leader of the Radical Party, has used this tool of nonviolent struggle on several occasions. Pannella has always linked his actions, in which he fed himself only with cappuccinos, to demand attention for the law by public authorities and for civil rights [48]. Before him, Danilo Dolci and Aldo Capitini used this instrument of political struggle in Italy [49]. Furthermore, Sardinian independence activist Salvatore “Doddore” Meloni died on 17 July 2017, after 66 days of hunger strike, in Uta prison (Cagliari, Italy) [50]. In summary, from ancient periods to the modern era, fasting has characterized the religious and political lives of humans. Beyond this, fasting became of medical interest at the beginning of 1900. One of the first scientists to apply fasting as a medical intervention was Herbert M. Shelton (1895–1985). During his career as a physician, he supervised more than 30,000 fasts between 1925 and 1970, providing evidence on the benefits of this practice on the human body. He wrote in one of his writings: ”Fasting must be recognized as a fundamental and radical process that is older than any other mode of caring for the sick organism, for it is employed on the plane of instinct and has been employed since life was first introduced upon the earth. Fasting is nature’s own method for ridding the body of diseased tissues, excess nutrients, and accumulations of waste and toxins” [51,52,53]. During the same historical period, several scientific approaches were conducted to evaluate the effect of reduced caloric intake and undernutrition on living organisms. The pioneering work of McCay on the effect of CR demonstrated a significant impact on longevity and lifespan [54,55]. This provided the basis for future nutritional interventions based on CR in all aspects until IF. This narrative and fascinating history of the involvement of McCay in investigating CR was nicely reviewed by McDonald and Ramsey [56].

During the years, to improve the benefits of IF, including therapeutic outcomes, different methods were described and detailed in the next paragraph.

### 1.2. A General View of IF Approaches

Because of the success of nutritional interventions based on CR in weight loss, different approaches to reducing caloric intake have been described with the aim of improving the benefits to body weight and increasing patient compliance, mainly through long-term interventions. One such approach is IF, which is defined as a period of fasting combined with days of ad libitum eating and has gained popularity as an alternative to CR [57]. This latter therapy, which has been used for over 10 years, is the gold-standard therapy for weight reduction in patients with obesity [58]. Nevertheless, as previously mentioned, a lot of patients experienced significant difficulties in properly adhering to a continuous CR nutritional plan due to daily limitations in food consumption [59]. Accordingly, it has been observed that adherence to continuous CR declines after approximately 1 month of intervention and continues to decrease afterward [7,60,61]. Considering this significant issue in the CR dietary regimen, different IF approaches in which fasting and feeding are alternated have been recently proposed based on some positive health benefits observed [4]. In fact, a recent meta-analysis of randomized clinical trials conducted by Gu and coworkers showed that nutritional interventions based on IF produced positive effects on most of the considered outcomes (weight, body, BMI, waist circumference (WC), fasting glucose, and triglyceride levels. In this study, the scientists considered each type of IF regimen (types of IF are reported in Table 1) applied in randomized clinical studies. This criterion of inclusion allowed to analyze 43 randomized controlled trials with a total of 2483 participants (1277 intervention group; 1206 control group) with an intervention time of at least 1-month (median 3 months). CR (continuous energy restriction, a Mediterranean diet, and Dietary Approaches to Stop Hypertension (DASH)) and non-intervention diet (the usual diet of the subjects without any modifications) were the eating patterns in the control group. Results showed some positive impact of IF in reducing body weight, WC, and fat mass, with no effects on lean mass compared with non-intervention diet. Again, IF improved blood lipid conditions and insulin resistance compared with a non-intervention diets. However, in this meta-analysis, IF did not show a superior profile with respect to CR for the outcomes considered. Furthermore, not all men and women, or the overweight or obese population, experienced the same effects of IF [62]. Interestingly, the only characteristic in women that was shown to be significantly lowered after IF was found to be the fat mass parameter, suggesting that IF may have little influence in this sub-population. In contrast, it was discovered that IF dramatically decreased triglyceride levels and weight in males. Unfortunately, the authors of the aforementioned study were unable to qualitatively analyze the energy intake of men and women because of different randomized clinical trial settings, mainly involving CR. Presumably, although it is not perfectly understood why this gender-based difference occurred, the gaps between males and females in energy intake, differences in the distribution of fat around the body, and sex hormones could play crucial roles in different responses to nutritional interventions [62]. Another meta-analysis considered different IF approaches with respect to the previously presented studies, and the effects on reducing body weight were compared with those of the classical CR. Elortegui Pascual and coworkers included, in their analysis, 24 randomized controlled trials for a total of 1768 individuals, which were suitable based on the PICO inclusion criteria [63]. The clinical studies retrieved were analyzed using random effect network analysis. They obtained results similar to those of the studies conducted by Gu and collaborators. In fact, based on the discussed meta-analysis, it can be concluded that IF is a viable weight loss approach and equivalent to CR, although some differences were observed related to different IF approaches [64]. Similar results were obtained from Schroor et al. in a meta-analysis in which 28 randomized clinical trials (*n* = 2043) were included, and the outcomes considered were anthropometric and cardiometabolic risk markers in healthy adults. Compared with CR diets, the IF dietary regimen did not produce significantly better improvements in anthropometrics and cardiometabolic risk indicators. However, there were larger decreases in WC and fat-free mass [65]. In addition, considering the specific population, the results did not differ from those previously presented. In fact, a recent meta-analysis using a random-effects model, conducted by Cheung and collaborators, in which the Chinese population was considered, demonstrated heterogeneity n the considered outcomes [66]. In particular, based on the proposed inclusion criteria, the authors considered nine randomized controlled trials with a total of 899 individuals, and the IF dietary regimen applied for at least 3 weeks in the intervention groups (different types of interventions) was compared with controls (ad libitum diet and CR). By comparing the obtained results with the control groups, the researchers found a significant reduction in body weight (−2.61 kg compared with control, ad libitum diet; −1.40 kg compared with CR group; overall −2.20 kg with respect to both control groups), BMI (−1.37 kg/m^2^ compared with control, ad libitum diet; −0.55 kg/m^2^ compared with CR group; overall −1.07 kg/m^2^ with respect to control groups), fat mass (−1.55 kg with respect to control groups), LDL-C (−4.11 mg/dL compared with ad libitum group; −0.01 mg/dL compared with CR), and triglyceride levels (−2.22 mg/dL com-pared with ad libitum control group; −5.94 mg/dL compared with CR). In contrast, WC (−2–12 cm over both controls), total cholesterol levels (−1.43 mg/dL compared with ad libitum control group; +2.69 compared with CR group; −0.63 mg/dL considering both controls), blood pressure (systolic and diastolic pressure values −1.99 mmHg and −1.84 mmHg, respectively), fasting glucose and insulin resistance (homeostasis model assessment of insulin resistance, HOMA-IR) (glucose levels −8.42 mg/dL with respect to the ad libitum control group; but no positive results vs. CR +1.65 mg/dL; HOMA-IR −0.48 with respect to control groups), HDL-C (−0.92 mg/dL compared with ad libitum group; +0.88 mg/dL compared with CR) were not significantly affected by the IF intervention. Adherence to the nutritional intervention was high, ranging from 84% to 97.5%. No serious adverse effects were reported globally. In conclusion, the studies confirmed that IF could be useful in reducing weight, whereas IF could not be helpful in improving some cardiometabolic parameters, including blood pressure [66]. This contrasted with a previous meta-analysis (694 individuals from different countries), in which a similar trend was observed, but the authors indicated a significant reduction in systolic blood pressure (−4.15 mmHg) [67], while longer IF interventions were found to positively modify the pressure outcome with statistically significant results (one-year observational study, 1422 individuals; −10.9 mmHg for systolic pressure and −5.8 mmHg for diastolic pressure). However, the discussed study has limitations. The main was the fact that this was an observational cohort study, which is known to have limitations when it comes to interpreting efficacy, and one year is not a sufficient long-term period to reach a convincing conclusion [68]. A more inclusive meta-analysis was conducted by Kim and collaborators, analyzing randomized controlled studies from 2011 to 2021 in which IF (all approaches based on IF) and CR were compared. Eligibility criteria accounted for an inclusive analysis of 16 randomized trials for a total of 1438 individuals (BMI 24 45 kg/m^2^; 18–70 years old; interventions 12–52 weeks). Results showed that IF and CR similarly performed in reducing body weight, and additionally, no statistically significant differences were found in LDL-C, total cholesterol levels, BMI, body fat mass, or fat-free mass between the groups [69]. Remarkably, few analyzed studies reported limited mild adverse effects, mainly hunger, headache, constipation, and fatigue, with no severe adverse effects observed in most of the examined clinical trials. Further details of the common undesired effects among the different types of IF are provided in Section 2.3.

Although IF sounds good for use in weight reduction and management, with improved outcomes with respect to the non-interventional groups, its superior profile with respect to CR needs to be further investigated by designing long-term clinical trials, personalizing the appropriate IF interventions to maximize, where possible, the positive outcomes, and reducing shortcomings related to the application of different IF dietary regimens. Accordingly, because IF results in similar weight loss and metabolic improvement as CR, it could be a valid alternative to CR for improving patient compliance and adherence to the proposed nutritional intervention. On the other hand, neither IF’+s impact nor its attrition rate were greater than that of CR [70].

In particular, IF could represent an additional instrument for clinicians and patients to increase clinician-patient concordance. In fact, IF can become a valid pragmatic tool to be proposed, especially for patients with continuous working hours, such as healthcare workers, for whom there is no possibility of a lunch break. Therefore, IF becomes more sustainable not only for university students but also for all those who lead a hectic life in which stopping and eating CR does not allow them to live the moment in serenity. In this way, the person will be able to choose, in agreement with the clinician, their most suitable eating time window, which will certainly help positively influence the subject’s nutritional re-education. Furthermore, IF has been shown not to have an economic impact on subjects with limited resources, which is an incisive factor in maintaining a patient’s nutritional path [71,72]. IF can assist the patient’s journey during the diet, as various studies have shown weight loss in American patients without changing their diet composition (including, for example, the consumption of some portion of junk food). This interesting fact could lead to hypothesizing the use of IF as an early approach to make the patient adhere to the nutritional path and, for example, if necessary, adopt the CR dietary regimen to bring the patient to nutritional re-education. and finally, under-stand together with the patient his eating propensities in temporal terms, leading the patient to nutritional autonomy with divided meals or cut out on the patient time windows dictated by the IF that can be used as a long-term lifestyle. Accordingly, IF can be a preventative and effective lifestyle strategy for the health of individuals who are more inclined to eat at closer times than meals divided throughout the day.

In general, when applying IF during the period of restriction of caloric intake, only 25% of normal caloric intake is allowed. However, considering the diverse IF-based approaches proposed, the stage of restriction could differ between hours and days. Drinking water is often allowed. Table 1 summarizes the different approaches based on IF.

**Table 1:** Main dietary interventions based on IF approach [73].

IF Method	Features	References
ADF Alternate day fasting	A day of eating is alternate to a day ad libitum is alternate to a day of fasting (25% of usual food intake, approximately 500 kcal)	[74]
IF 5:2	A five days with normal eating and two days of severe fasting (food intake restricted to 500–800 cal)	[64]
FMD Fasting-mimicking diet	A five-day fasting dietary regimen centered around natural, healthful items and ingredients (healthy fats and fiber-rich carbohydrate) with no refined carbohydrate (25% of usual food intake and less than 10% of protein)	[75,76]
PF Periodic fasting	Water-only fasting or FMD for at least two days in succession repeated each month (involves a maximum daily energy intake of 250 kcal for about one week–5-day diet affording 750–1100 kcal)	[77]
TRF Time-restricting feeding	Daily no energy intake (or restricted amount) for 12–20 h, with eating window of 4–12 h (reduction of at least 20% of caloric intake)	[78]
eTRF Early time-restricting feeding	Modified TRF in which calories restriction occurs in the first 6–8 h of the day (i.e., eating window starts at 08:00 a. m. to at the maximum 02:00 p.m.)	[79]
lTRF Late time-restricting feeding	Modified TRF in which the eating window starts late in the day, usually from 02:00 p.m. to at the maximum 08:00 p.m.)	[80]

#### 1.2.1. ADF-Alternate Day Fasting and 5:2 Diet

All varieties of IF involve fasting intervals that are sustained over a typical night-long fast of 8–12 h [81]. Among the different approaches reported in Table 1, alternate day fasting (ADF) is one of the most used and studied IF approaches. This strategy for reducing calorie intake involves alternating feasting days on which individuals have no restrictions on the types or quantities of foods consumed over 24 h, with fasting days on which individuals are required to limit their caloric intake. In this case, individuals can choose to consume 25% of their energy needs (approximately 500 kcal per day), which is called modified ADF, or alternatively, they can consume only water, which is termed zero-calorie ADF. This more extreme version of the diet requires individuals to fast completely for a 36 h period and feast only during a 12 h window. The fast-day meal can be consumed all at once or spread throughout the day, and the timing throughout the day is optional. Interestingly, accumulating data indicate that not only are fasting periods per se important for maintenance and improvement of metabolic health but also the timing of meals matters, favoring early intake of calories rather than late in the evening [82], although participants generally prefer to consume the meal at dinner time so they can engage in their habitual social eating patterns [4,83,84,85,86,87]. Furthermore, ADF, compared with CR, could exhibit superior compliance with respect to CR [10,88], lacking the burden of persistent nutritional deprivation and other negative effects [5]. In dramatic contrast, other studies have reported that ADF could not be a possible dietary regimen because of extensive and constant hunger, as reported in different studies [89,90]. Surely, hunger could negatively modify the adherence and enthusiasm of individuals involved in fasting. Considering the importance of ADF, we have dedicated a section in which several clinical studies were analyzed in depth and different ADF-based approaches were investigated.

A modified version of IF is represented by the 5:2 diet, which involves a fast of 24 h twice a week and five feasts on other days per week. Fast days can occur on consecutive or non-consecutive days throughout the week [84]. The 5:2 diet is a simple and efficient method for reducing weight and enhancing metabolic health. It is far easier for many individuals to follow than a traditional diet involving CR. The strategy has an added benefit in that, should it prove successful, underprivileged populations may benefit most from it [91,92]. The majority of weight management regimens currently in use contain complicated information about diet, food composition, coping mechanisms, behavior, food journals, exercise, and other topics. In addition, they typically require significant lifestyle adjustments and incur expenditures, such as those associated with commercial diet substitutes. Even for those who possess substantial socioeconomic means and lead orderly lives and routines, all of these needs might be challenging to comprehend and execute. Participants from middle-class to upper-class backgrounds are included in the majority of studies in this area, and the results usually show poor adherence and very moderate weight loss. Those with high stress levels, a high frequency of unforeseen events, and limited resources may find 5:2 particularly promising because it is considerably simpler and less demanding. However, even 5:2 places a lot of pressure on fasting days, and its overall adherence may be just as low as that of other regimens [93]. Hajek and colleagues analyzed the effect of a 5:2 diet on a population recruited from a city zone with high deprivation by conducting a randomized controlled trial (ISRCTN79408248). They enrolled 300 obese participants (adults, BMI ≥ 30 kg/m^2^ (or ≥28 kg/m^2^, with co-morbidities) and then randomly divided them into three different groups and 1 year follow-up [control group, Standard Brief Advice (SBA)-diet and physical activity (*n* = 100); 5:2 self-help instructions (5:2SH) (*n* = 100); or 5:2SH plus six once-weekly group support sessions (*n* = 100). Results indicated that adherence was significant during the first 6 weeks (74%), whereas a dramatic decline after six months (31%) and one year was observed (22%). Furthermore, after 6 months, 5:2SH and SBA achieved similar modest results in reducing weight (−1.8 kg and −1.7 kg, respectively). Also, the analyses at one year were comparable. Interestingly, the 5:2SH group who received group support sessions showed favorable value regarding weight loss with respect to the 5:2SH group without group support sessions at six weeks (−2.3 kg vs. 1.5 kg). After one year, statistically significant results were obtained. In summary, the introduction of a group support session could improve the efficacy of the intervention and should be adopted, especially in areas where deprivation is high [93]. A small non-blinded randomized controlled clinical study (NCT04319133) evaluated the effects of a 5:2 diet for eight weeks on different body parameters in 50 subjects presenting BMI  ≥  25 kg/m^2^, a WC of 90 cm, and fasting blood glucose  <  125 mg/dL (at each group, control (no fasting without restriction) and intervention (5:2 diet) were assigned to 25 participants). Results indicated that during the 8-week intervention period, there was no discernible difference between the non-fasting and fasting groups in terms of the mean change in BMI, muscle mass, fat mass, and % body fat. In the intervention group, there was a slight variation in body weight. Unfortunately, no comparison with CR can be performed because there was not a CR group as a control [94]. These findings contrasted with a recent study conducted by Fudla and colleagues in which 40 obese male students (age 18–25 years old) were enrolled in a randomized clinical trial and completed the investigation with a fasting compliance score of ≥85%. Individuals were randomly assigned to the intervention group (*n* = 20) and control (*n* = 20). The control group continued to eat as usual and recorded their consumption using a three-day, 24 h recall, whereas the intervention group fasted for two nonconsecutive days each week and kept a food diary. Following a 4-week fast, there was a statistically significant difference in the reduction in energy intake and BMI between the control and intervention groups. Individuals in the intervention group showed a reduction in their BMI of approximately 0.47 kg/m^2^ (*p* < 0.05) and an increase in calorie consumption of approximately 406.68 cal (34.6%) with *p* < 0.001. In contrast, the control group showed a drop in BMI of 0.11 kg/m^2^ and an increase in energy intake of approximately 321.73 cal. A similar trend was observed when considering fat intake [95]. Moreover, the effects of the IF 5:2 intervention were evaluated in a randomized clinical study (IRCT20100524004010N31) in patients affected by non-alcoholic fatty liver disease (NAFLD) compared with a control group (no fasting, usual dietary regimen). Kord Varkaneh and colleagues used a computer-generated random-numbers approach to randomly assign eligible persons with NAFLD (*n* = 24) to either the IF (5:2) group or the non-interventional control group (*n* = 25) (no modification with respect to the usual dietary regimen) and followed them for twelve weeks. The participants were first stratified based on their age and BMI, respectively (BMI = 25–40 kg/m^2^, age 18–50 years old, NAFLD-grade 2). In this case, the results presented discrepancies in the statistically significant outcomes. In fact, weight loss, anthropometric obesity indicators, and parameters related to the NAFLD condition (liver enzymes, inflammatory biomarkers, hepatic steatosis, and triglyceride levels) were significantly reduced after the dietary 5:2 intervention. Other important parameters, including HDL-C, LDL-C, total cholesterol, insulin, fasting blood sugar, and HOMA-IR, were not statistically affected by the proposed dietary intervention. Unfortunately, no control group assigned to CR was present, and accordingly, no direct comparison can be performed. Results were in line with the higher degree of discrepancies found by applying the IF 5:2 diet, although, interestingly, the use of IF in individuals suffering from a specific metabolic disorder such as NAFLD significantly reduced the parameters related to the pathological condition [96].

#### 1.2.2. TRF—Time-Restricting Feeding (eTRF and lTRF)

Time-restricting feeding (TRF), also known as time-restricted eating (TRE), is another popular approach in which fasting occurs every day with variable hours; it is a unique form of IF in that it does not require individuals to monitor their energy intake or to count calories during the eating window [97]. In fact, it involves confining the eating window to a specified number of hours per day (typically 4 to 8 h) and fasting with water or zero-calorie beverages for the remaining day, allowing the duration of the fast to be 14–18 h [4,84]. For example, an illustration of TRF is if you decide to eat all your food for the day in a period of 8 h, such as from 10:00 a.m. to 06:00 p.m., the remaining 16 h represent the fasting period, during which no calories should be consumed. Unfortunately, at present, there are no large clinical studies on this novel dietary intervention [98,99]. However, some indications could be extracted by analyzing the described outcomes. Moro and colleagues enrolled 34 male resistance-trained subjects, who were randomized to receive either a normal diet (100% of their energy divided into three meals consumed at 08:00 a.m., 01:00 p.m., and 08:00 p.m.) or TRF and followed for 8 weeks of intervention. Interestingly, the related findings highlighted that resistance training combined with an IF regimen (TRF), where all calories are ingested within an 8 h window each day, may enhance certain health-related indicators, reduce fat mass, and preserve muscle mass in male resistance-trainers. This suggests that TRF could be used by athletes during their maintenance periods of training, when the objective is to retain muscle mass while decreasing fat mass, although larger interventions are necessary to confirm the reported findings [98]. Gabel and colleagues conducted the first clinical trial (NCT02948517) that scrutinized the impact of TRF in an obese population. They investigated the effects of an 8 h food restriction (TRF) on body weight and metabolic disease risk factors in obese individuals after 12 weeks of treatment. Individuals with a BMI ranging from 30 to 45 kg/m^2^ and aged between 25 and 65 years were selected and randomized into an intervention group (*n* = 23; TRF 8 h eating window; ad libitum 10:00 a.m.–06:00 p.m., fast 06:00 p.m.–10:00 a.m.) or control group (*n* = 23; usual habits and diet). Results indicated that the TRF regimen caused a mild reduction in weight and energy intake (−2.6% and −341 kcal/day, respectively). Several parameters were not significantly affected by the dietary regimen. In fact, parameters such as fat mass, triglyceride levels, LDL-C, HDL-C, diastolic blood pressure, HOMA-IR, fasting insulin, and fasting glucose levels were not significantly modified by the proposed dietary regimen. In contrast, a significant reduction in systolic blood pressure (−7 mmHg) was observed [99]. A subsequent prospective randomized clinical study conducted by Lowe and coworkers corroborated the previously discussed findings on the benefits of TRF in reducing body weight (NCT03393195 and NCT03637855). The researchers focused their attention on the following question: for individuals who are overweight or obese, what impact does TRF have on weight loss and metabolic health? To answer this question, 116 participants were enrolled (18–64 years old), living in the USA, and sex-balanced with a BMI ranging from 27 to 43 kg/m^2^. They were divided into two groups: one control group (three structured meals per day) and one intervention group trained to eat as much as they wanted between 12:00 p.m. and 08:00 p.m. and to absolutely avoid eating anything from 08:00 p.m. until 12:00 p.m. the next day. The duration of the intervention was fixed at 12 weeks. Results showed a modest weight reduction in the TRF group (−0.94 kg; 95% CI), with no significant changes in the control group (−0.68 kg; 95% CI). However, no significant differences were observed between the two groups (−0.26 kg; 95% CI). Furthermore, they did not observe improvements in the secondary outcomes (fat and lean mass, fasting insulin and glucose, glycated hemoglobin (HbA1c) levels, estimated energy intake) including the estimated energy intake. In conclusion, this type of TRF did not improve the reduction in body weight compared with normal eating during the day. However, there is not a direct comparison with CR, and the study presents some limitations, including the absence of measurement of calorie intake and changes in protein intake in the intervention group and lack of self-reported data on energy or macronutrient intake. On the other hand, randomization, practical prescription-based intervention, and a suitable control group were some of the study’s strong points [100]. Finally, a recently published systematic review reported the same conclusion by comparing TRF and CR. Ezzati and colleagues analyzed seven randomized clinical trials (*n* = 579) in which TRF was compared with CR and discussed outcomes in relation to CR alone. The authors considered a duration of the interventions ranging from 8 to 52 weeks, in which patients with overweight and obesity (32–58 years old belonging to different countries USA, Turkey, Taiwan, China, and Australia, with a BMI > 26 kg/m^2^). The results showed that, at least for the majority of the selected studies, there were no appreciable variations in weight loss between TRF and CR, and only one study indicated that a weight reduction of 4.1% was achievable in obese patients with TRF with respect to CR (2.1%). No significant differences in secondary outcomes (WC, body fat and lean mass, total cholesterol, fasting blood glucose, HOMA-IR, HDL-C) were detected between TRF and CR. In contrast, improvements in blood pressure parameters and LDL-C levels were more significant in the TRF group than in the CR intervention group, in accordance with previous studies [101]. Accordingly, although no significant variations were observed in lowering body weight and improving some secondary outcomes considering the TRF dietary regimen vs. CR, further investigation of the different TRF dietary regimens could be important for establishing differences among nutritional interventions to enhance the efficacy of the TRF approach. In light of this, Cienfuegos and coworkers conducted the first clinical trial (NCT03867773) to evaluate different TRF regimens (4 h and 6-h) on 58 obese patients (BMI 30–50 kg/m^2^). The 4 h TRF group (*n* = 19) was given instructions to fast from 07:00 a.m. to 03:00 p.m. (20-h fast) and eat ad libitum from 03:00 p.m. to 07:00 p.m. every day for the 8-week intervention. The 6 h TRF group (*n* = 20) was told to fast from 07:00 a.m. to 01:00 p.m. (18 h fast) and eat whenever they pleased from 01:00 p.m. to 07:00 p.m. every day. The control group (*n* = 19) was established, and patients did not receive nutritional indications. TRF participants were not asked to track their calorie intake during the feeding windows, nor were there any restrictions on the kinds or amounts of food that they could eat. While black tea, coffee, and diet soda are energy-free beverages that can be consumed throughout the fasting window, the TRF participants were encouraged to drink a lot of water. Compared with controls, 4- and 6 h TRF led to similar decreases in body weight (about 3%), insulin resistance, and oxidative stress after 8 weeks. Without tracking calories, energy intake decreased by approximately 550 kcal/day in both TRF groups. These results imply that 4- and 6 h TRF cause modest decreases in body weight over the course of 8 weeks, indicating their potential as weight loss therapies. In addition, these diets may enhance certain elements of cardiometabolic health [102]. A randomized clinical trial was conducted by Liu and colleagues to evaluate the performance of TRF over classical CR (NCT03745612). They enrolled 139 patients (BMI 25–45 kg/m^2^; 18–75 years old) with no significant disorders related to overweight and obese conditions who did not participate in weight-loss programs. Individuals were randomly assigned to the TRF (*n* = 69) or daily CR (*n* = 70) groups and followed for one year. Results showed that adherence to the trial was relatively high (84.0 ± 16.1% and 83.8 ± 12.6% considering TRF and CR groups, respectively). Notably, considering the interventional period of 12 months, there were no significant differences in the two groups’ average caloric deficit or the proportions of calories from fat, carbs, and protein. No statistical differences were found in body weight reduction between the two groups (TRF −8.0 kg and CR −6.3 kg; net differences −1.6 kg). In both groups, the proportions of individuals who had lost more than 5%, 10%, and 15% of their body weight at 12 months were comparable. Furthermore, individuals in both groups experienced comparable decreases in their baseline BMI (TRF −2.9 kg/m^2^ and CR −2.3 kg/m^2^) and WC (TRF −8.8 cm and CR −7.0 cm). Notably, the reduction in body composition parameters was comparable between the two groups, with no observed changes in the amount of lean mass, visceral fat in the abdomen, subcutaneous fat, and liver fat lost by the TRF and CR diet groups. Furthermore, over a 12-month period, TRF and CR were linked to lower systolic (TRF −8.1 mmHg and CR −7.7 mmHg) and diastolic (TRF −5.1 mmHg and CR −3.8 mmHg) blood pressure, with no discernible group difference. Finally, fasting glucose levels, 2 h postprandial glucose levels, HOMA–IR, and lipid levels were comparable between the two selected groups during the period of the study. As previously mentioned for other studies, during the investigated trial, no relevant adverse effects (death or severe undesired effects) were detected, whereas minor undesired effects, including headache, appetite decrease, dizziness, fatigue etc. were found to occur with similar frequency in both groups [103]. The same conclusion was reached by Lin and colleagues by analyzing the results of a randomized clinical trial (NCT04692532), also considering different racial populations. They enrolled 90 obese patients (BMI 30–50 kg/m^2^; 40 years old median age; Black 33%; Hispanic 46%; white 21%), and 77 completed the study. After 12 months of intervention, the results showed a comparable reduction in energy intake among the TRF and CR groups (−425 kcal per day and −405 kcal per day, respectively) and in lowering body weight (control group, consuming food for 10 h or more every day: −4.61 kg, *p* ≤ 0.01; TRF group, merely consuming food from noon to 8:00 p.m., without monitoring calories: −4.87%;CR group, 25% energy restriction daily: −5.30%, *p* ≤ 0.01). Interestingly, no significant differences were observed between TRE and CR considering the different racial populations between TRF and CR (0.81 kg, 0.43%). However, the study has some limitations: it was not powered to detect reasonably large variations in weight reduction, not blinded, and not multiple comparison adjusted [104]. In summary, this further presented study confirmed a similar impact of IF and CR dietary regimen in reducing body weight, while successfully both reduced the daily calorie consumption, experiencing an identical success rate in obese patients by applying both nutritional interventions. In contrast, another trial indicated the greater effectiveness of TRF over CR in reducing body weight in patients with type 2 diabetes mellitus (T2DM). In fact, in a 6-month randomized clinical trial involving 75 individuals (HbA1c levels 6.5–11.0%, age 18–80 years old, and BMI 30–50 kg/m^2^) suffering from T2DM, they were randomly divided 1:1:1 into three groups: TRF (ad libitum 12:00 p.m. and 8:00 p.m. daily without monitoring caloric intake with no restriction on foods and fasted from 8:00 p.m. to 12:00 p.m. the following day, in which individuals were encouraged to drink abundant water, allowing energy-drink consumption), CR (reduction in energy intake by 25%), and control (usual habits and eating. In comparison with controls, the TRF group’s body weight had considerably dropped by month six (−3.56%), but not in the CR group (−1.78%). Compared with controls, HbA1c levels dropped in the TRF (−0.91%) and CR (−0.94%) groups without significant differences. Blood pressure, plasma lipid levels, medication effect score, and time in the euglycemic range were all the same in all groups [105].

As reported in Table 1, the TRF can be modified on the basis of the adopted eating windows. In particular, if the eating widows with no restrictions on feeding occur in the early hours of the day, the approach is known as early TRF (eTRF), whereas if the eating window occurs later in the day, the TRF is called late TRF (lTRF). Are there indications for the most efficacious TRF methods? Recently, a preclinical study showed that mice fed a high-fat diet for 14 weeks, followed by a nutritional intervention based on eTRF or lTRF for 8 h for 5 weeks, were more susceptible to reducing body weight and improving metabolic state when eTRF was adopted. In particular, animals were divided into control groups (ad libitum high-fat diet or low-fat diet, *n* = 24) vs. animals treated with eTRF (*n* = 24) and lTRF (*n* = 24). Results showed that, in comparison to mice fed the lTRF diet and control group (ad libitum high fat diet), eTRF resulted in reduced body weight and fat depots, as well as lower levels of insulin, glucose, cholesterol, C-peptide, TNFα, leptin, and alanine aminotransferase. Compared with the control groups, in the TRF diet, both eTRF and lTRF showed a significant decrease considering inflammatory and fat formation phenomena, whereas eTRF was associated with advanced hepatic circadian rhythms with higher amplitudes and clock protein expression levels. In summary, TRF enhanced the metabolic status of muscular and adipose tissues. In particular, compared with high-fat diet-fed mice, but similar to low-fat diet-fed mice, eTRF leads to reduced body weight, lipid profile, and inflammation in addition to enhanced insulin sensitivity and fat oxidation. These statistics emphasize the importance of timing meals compared with ad libitum food, especially during the first several hours of the activity period [106]. The effects of eTRF and lTRF in humans were evaluated in one of the first clinical trials (randomized crossover trial NCT02633722) designed for this purpose. Hutchison and coworkers investigated the effects of 9 h TRF, eTRF, or lTRF on glucose tolerance in men at risk for T2DM. In two 7-day TRF conditions and for 7 days of baseline assessment, fifteen men (BMI 33.9 ± 0.8 kg/m^2^, age 55 ± 3 years old) were monitored with a constant glucose monitor. There was a 2-week washout period between the participants’ randomization to either eTRF (08:00 a.m.–05:00 p.m.) or lTRFd (12:00 p.m. to 09:00 p.m.). As a result, regardless of when TRF is started (eTRF or lTRF), this study has shown that in men at risk for T2DM, a week of TRF improves glucose responses to meals [107]. Similarly, Sutton and coworkers investigated the effects of TRF on oxidative stress, blood pressure, and insulin sensitivity in prediabetic men in a 5-week, randomized, crossover, isocaloric, and eucaloric controlled feeding trial. They enrolled several individuals, but unfortunately, only eight men completed the trial. In any case, they (aged 56 ± 9 years old) showed a mean BMI of 32.2 ± 4.4 kg/m^2^, fasting insulin of 25.1 ± 14.5 mU/L, fasting glucose of 102 ± 9 mg/dL, and a 2 h glucose tolerance of 154 ± 17 mg/dL. Blood pressure and mean lipid levels were within the standard ranges. Results showed that in males with prediabetes, eTRF reduced blood pressure, oxidative stress, insulin sensitivity, and insulin levels, even though food intake was the same as that in the control group, and no weight loss occurred. This study was the first randomized controlled experiment to demonstrate the benefits of IF for human weight loss and food intake [108]. To confirm this conclusion, a larger cohort with more strictly controlled free-living periods should be included in this experiment. In general, large-scale, long-term randomized controlled trials are necessary because of the ease of use of TRF and its effectiveness in improving glycemic outcomes. Recently, a meta-analysis was conducted to investigate which eTRF and lTRF practices could produce more beneficial effects on the human body. To shed some light on this controversial discussion, Liu and coworkers analyzed 12 randomized clinical trials including 730 obese or overweight individuals using a network meta-analysis to evaluate the impact of both TRF dietary interventions on blood pressure, lipid profiles, glycemic metabolism, and body weight. Compared with non-TRF, eTRF and lTRF both produced moderate reductions in body weight and insulin resistance. It is interesting to note that improvement in insulin resistance was more successful with eTRF than with lTRF (eTRF vs. lTRF: −0.44; *p* < 0.05), although there was no statistically significant variation in weight loss (eTRF vs. lTRF: −0.31 kg; *p* > 0.05). Furthermore, compared with non-TRF, eTRF rather than lTRF showed significant advantages in blood pressure and glucose metabolism. Lipid profiles, blood pressure, and fasting blood glucose levels were not significantly altered between eTRF and lTRF. According to the meta-analysis, there was no relevance to which methods could be chosen to effectively manage weight and obtain metabolic benefits [109]. Interestingly, Xie and colleagues investigated the effect of different approaches to TRF on health benefits in non-obese individuals with no diseases. In order to examine the effects of the two TRF regimens in healthy adults who were not obese, a 5-week randomized trial was conducted (ChiCTR2000029797). Using a computer-based random-number generator, 90 participants were randomized to either the eTRF (*n* = 30), mild-TRF (mTRF) (*n* = 30), or control groups (*n* = 30). After completing the five-week experiment, eighty-two participants, 28 in the eTRF, 26 in the mTRF, and 28 in the control groups, were evaluated. The alteration in insulin resistance was the main result. Although participants and caregivers were not blinded to group assignment, researchers who evaluated the results were. Interestingly, eTRF improved insulin sensitivity more than mTRF. In addition, eTRF, but not mTRF, improved fasting glucose, decreased BMI and obesity, reduced inflammation, and boosted the variety of microbes in the gut. Throughout the experiment, no significant adverse events were recorded. In summary, eTRF outperformed mTRF in terms of improving insulin resistance and associated metabolic markers.

According to the findings in the mentioned studies, an interesting clinical study (ISRCTN32122407) was registered by Lynch and coworkers with the aim of evaluating, by comparing the effects of eTRF vs. lTRF, changes in metabolic disease risk factors and their impacts on social well-being and quality of life in a group of persons who self-assess to be at elevated risk of developing T2DM. The authors will evaluate different parameters, including modifications to (i) insulin resistance using the HOMA-IR method, (ii) LDL-C levels in plasma, (iii) body weight and composition, (iv) dietary energy intake, (v) metabolic disease risk factors (i.e., blood pressure, oral glucose tolerance, fasting plasma levels of lipids and glucose). Eligible individuals aged 18–65 years with a BMI ≥ 25 kg/m^2^ were enrolled in this study and randomized into eTRF (7:00 a.m. to 03:00 p.m.), lTRF (12:00 p.m. to 08:00 p.m.), or control group (unlimited eating window) and followed for 10 weeks. Despite the interesting outcomes to be discussed, at the moment of writing this review article, the results have not been published because the study was recently completed and the related publications were planned for April 2024 [80]. A similar study was conducted on 90 obese adults (80% female; mean BMI, 39.6; mean age, 43 years old; NCT03459703). The subjects were randomized in three groups with different energy-restricted weight-loss treatments: (eTRF (8 h eating 07:00 a.m. to 03:00 p.m.), control group (≥12 h window with energy restriction). Results showed that, compared to eating throughout a period of 12 or more h at 14 weeks, eTRF was more effective for weight loss, diastolic blood pressure improvement, and mood enhancement [110].

To induce further darkness in the IF dietary approach, and, in particular, on the 8 h TRF dietary intervention, a communication last month at the “Epidemiology and Prevention Lifestyle & Cardiometabolic Health” meeting organized by the American Heart Association reported the results of a study that is highly controversial with respect to the presented ones. In particular, Chen and Zhong, in a communication titled “P192-Association Between Time-Restricted Eating and All-Cause and Cause-Specific Mortality” reported the results of a large observational study to understand the long-term effects of TRF. They compared data from the Centers for Disease Control and Prevention’s National Death Index database, which contains information on deaths in the United States from 2003 to December 2019, with information regarding dietary patterns for participants in the annual 2003–2018 National Health and Nutrition Examination Surveys (NHANES). The study included 20,078 individuals who underwent 8 h TRF. Surprisingly, the outcomes of the study showed that eating for less than 8 h was substantially linked to a higher risk of cardiovascular death (HR, 1.96) compared with eating for 12–16 h. Individuals with cancer (HR, 2.72) and adults with cardiovascular disease (HR, 2.06) also showed this connection. Except for eating for 8–10 h or longer in patients with cardiovascular disease, other eating times were not linked to cardiovascular mortality (HR, 1.64). Eat for more than 16 h was linked to a lower risk of cancer-related death in individuals with cancer (HR, 0.46), but no other significant relationships were observed between eating duration and all-cause cancer mortality in the whole population or sick subsamples. Remarkably, TRF with a meal duration of less than 8 h was substantially linked to an increased risk of cardio-vascular mortality in the general population (91%), as well as in individuals with cancer or cardiovascular disease. These results contradict the long-term benefits of 16:8 TRF in reducing cardiovascular mortality [111,112]. Despite some limitations (i.e., reliance on self-annotated dietary data, which could affect the assessment of eating patterns), this study is in total disagreement with most studies reporting the benefits of TRF on healthy status. Obviously, several responses to the presented reports can be found on the web and raised several concerns about the robustness of the study, which lacks elements to conduct an accurate assessment of the results [113,114]. However, these findings require confirmation to propose a better dietary intervention based on the TRF approach.

According to the proposed discussion, we summarize the advantages and disadvantages of different IF approaches in Table 2.

## 2. Food Intake Restriction: An In-Depth Outlook

As previously discussed, IF refers to eating patterns that require the consumption of little or no calories for a period of time, typically a minimum of 12 h, followed by a period of ad libitum eating. IF has gained increasing popularity as an alternative to continuous CR because it does not require patients to vigilantly monitor energy intake or meticulously track calories every day, nor does it forbid individuals from eating certain food groups. Moreover, some IF regimens permit individuals to eat freely during certain periods of the day. Taken together, all these features make IF an attractive and simple lifestyle that is easy to incorporate into adult daily life. Research has mainly targeted two types of IF, namely ADF and TRF.

Recently, there has been increasing interest in improving dietary interventions through various nutritional regimens, such as IF, which has gained much public interest as a weight loss approach. Since fasting is known to stimulate adaptive cellular responses, including improved glucose regulation, increased stress resistance, suppressed inflammation, and autophagy upregulation, it is hypothesized that altering body metabolism will lead to long-term health benefits [1,115,116]. In the following paragraphs, the main IF protocols based on ADF will be analyzed through completed clinical trials aimed at investigating the potential health benefits of these approaches, whether they are used in obese, diabetic adults or healthy, non-overweight adults. In fact, as nicely reviewed by Varady and colleagues [117], ADF could represent a promising strategy for preventing diseases, including chronic diseases, by modifying the impact of different (chronic) disorder risk factors, as highlighted for the CR dietary regimen [118,119,120,121]. Accordingly, human [89,122,123] and animal studies [124,125] have provided evidence concerning ADF and the risk of certain chronic diseases, such as T2DM, cardiovascular and neurological diseases, and cancer. Considering the risk factors for T2DM, studies have indicated that the effects were comparable to those found by applying CR as a nutritional intervention. Specifically, ADF showed significant glucose uptake mediated by insulin, whereas the effects on insulin concentration and fasting glucose were not significant [123]. Regarding cardiovascular risk factors, ADF could lower total cholesterol and triacylglycerol levels, increasing HDL-C concentrations, whereas controversial results were reported considering blood pressure values [89]. There is currently no exhaustive information regarding the cancer risk in humans that can be improved by the ADF dietary regimen; however, research on animals has shown decreased incidence of lymphoma, longer survival following tumor inoculation, and decreased rates of proliferation of many proliferating cell types.

Lastly, a recent investigation regarded autoimmune diseases. It has been hypothesized that ADF could play a crucial role in improving the status of patients with autoimmune disorders such as multiple sclerosis, psoriasis, thyroid syndromes, and systemic lupus erythematosus. Unfortunately, the incomplete evidence from limited studies, which were inconclusive, did not allow the establishment of a validated efficacy of IF/ADF against autoimmune disorders. Given the importance of this topic related to ADF, further investigation is required to establish the best practices for IF and its long-term impact [126,127]. The following subchapter was developed to analyze IF as a therapeutic dietary intervention for common neurological disorders.

### 2.1. IF in Neurological and Psychiatric Disorders: Depression, Anxiety, Mental Health, and Neurodegenerative Diseases

Remarkably, some studies have evaluated the effects of ADF on neurological diseases such as epilepsy [128], resistant anxiety [129], and depression and psychiatric disorders [130]. Also, in this case, the results were not always significant, indicating that the dietary intervention did not negatively affect the psychiatric diseases in the selected patients. In effect, IF had a slight positive influence on diminishing depression scores, whereas no significant results were obtained for mood or anxiety.

#### 2.1.1. IF and Mental Health

In a secondary analysis of the previously discussed study [104], Lin and colleagues assessed for the first time different psychological/psychiatric parameters related to IF (TRF) and CR dietary interventions, such as Beck Depression Inventory II (BDI-II) [131], Profile of Mood States (POMS) [132], and quality of life by the Rand 36-Item Short Form (SF-36), to measure mental (vitality and mental health) and physical (bodily pain and physical health) aspects of quality of life [133]. According to the BDI-II survey results, the control and CR groups did not exhibit any discernible depression at baseline, whereas the TRF group experienced mild depression. By month 12, there was no difference in the TRF or CR group’s BDI-II depression score compared to the controls. In all groups, the baseline values of the POMS vigor (positive mood) scale were high, whereas those of the depression, anxiety, exhaustion, and confusion scales were low. In addition, all groups had modest baseline POMS total mood disturbance scores. These findings suggest that, at the start of the study, individuals in each group were generally in a positive mood. In comparison with the controls, neither the total mood disturbance score of the TRF or CR groups nor any of the POMS subscales changed by month 12. Regarding quality of life, the SF-36 categories measuring overall physical health, bodily discomfort, mental health, and vitality were all reasonably high in the TRF, CR, and control groups at baseline. These findings indicate that all groups had an overall high quality of life at the start of the study. After 12 months of intervention, no appreciable differences in mental health, physical pain, or overall physical health were observed between the TRF and CR groups and the controls. In contrast to controls, greater vitality was observed in the TRF group (7.77, 95% CI, *p* = 0.05). In summary, compared with the no intervention controls, the findings showed that 12-month treatment with TRF and CR had similar impacts on weight loss but no influence on mood, mental health, or quality of life [134].

Furthermore, the limited number of clinical studies evaluating the effects of IF on depression did not lead to conclusions. The effect of IF on depression symptoms is unclear. IF may function as a circadian regulator by enhancing neurotransmitter availability and elevating brain-derived neurotrophic factor (BDNF) levels, according to research on animal models. The human studies, on the other hand, were primarily conducted on healthy participants and revealed significant variation in the IF regimen examined as well as the observed effects on mood. The particular limitations of most clinical trials that are currently available include small sample sizes and uncontrolled designs. Although IF may have physiological effects that could be important in mood disorders, more thorough research and controlled trials are required to determine whether or not IF is effective in treating major depression, especially in psychiatric patients [135]. In fact, only two systematic reviews have been published to date [130,136], including a limited number of analyzed studies. In the first study, Fernández-Rodríguez and colleagues collected randomized and non-randomized clinical trials considering all types of IF interventions. They analyzed 14 studies (8 randomized and 6 non-randomized) (*n* = 562). They indicated that IF had a moderate effect on depression scores compared with the control groups, whereas no significant effects of IF on mood and anxiety were observed. IF modalities did not negatively impact mental disorders in the general population. IF had a moderate influence on decreasing depression scores, with no significant modification in anxiety and mood [130]. The other studies conducted by Berthelot and colleagues collected 14 clinical studies, including observational studies or controlled trials (*n* = 1436), in which every type of fasting was considered, including Ramadan. Results indicated modest improvement in anxiety and depression symptoms, and these preliminary findings should be taken with a grain of salt, considering the several limitations of the study. In fact, psychiatric populations should be the subject of more trials as they would be suitable candidates for fasting therapies; no study was conducted in this area. Furthermore, no studies in comparison with CR were present there were just 11 studies total, most of which had small sample sizes. Two controlled trials on IF were non-randomized, and four observational studies were conducted during Ramadan. Accordingly, the sensitivity was low due to the insufficient number of studies [136]. The same findings were highlighted in a recently published systematic review (June 2024) by Sharifi et al., which aimed to investigate the effects of IF and TRF on cognitive function and mental health in elderly people. They collected 8 clinical trials (*n* = 4006), including pilot trials, cross-sectional, observational, and cohort studies, experimental design, and a randomized controlled trial. The results of these different types of investigations are not always in agreement. The improvement in mental health status was modest and nonsignificant, as previously reported, whereas some improvement in cognitive function was observed. However, the previously reported limitations also affected this systematic review, and the conclusions should be taken with caution [137]. In fact, several better-designed clinical trials should be conducted to effectively assess the impact of IF on mental health and the related main psychiatric disorders, such as anxiety and depression.

#### 2.1.2. IF, Cognitive Function, and Neurodegeneration

Concerning the improvement of cognitive functions related to IF and CR, the influence of these dietary regimens seems to be more impacted with respect to the outcomes observed for mental health and neurological disorders. In fact, after relevant evidence in preclinical investigations [138,139,140,141], in the first clinical study on this field, Witte and coworkers conducted a prospective interventional clinical trial to evaluate possible improvements in cognitive function after CR dietary intervention. For this purpose, they enrolled 50 healthy elderly adults ranging in weight from normal to overweight (mean age 60.5 years old, mean BMI 28 kg/m^2^) and divided them into three groups based on their health: (a) calorie restriction (30% reduction), (b) relative increased intake of unsaturated fatty acids (UFAs) (20% increase, unaltered total fat), and (c) control. Following CR for 12 months, verbal memory scores significantly increased (mean increase 20%; *p* < 0.001), which was correlated with lower fasting plasma levels of insulin and high-sensitive C-reactive protein. These correlations were strongest in subjects who adhered to the diet the best. No observable changes in memory were observed in the other two groups, with no change in BDNF levels. This interventional study showed that CR can improve memory function in older, healthy participants. Higher synaptic plasticity and neurofacilitatory pathway stimulation in the brain due to enhanced insulin sensitivity and decreased inflammatory activity could be the mechanisms behind this improvement [142]. In a subsequent randomized clinical trial (NCT01286389), Horie and colleagues enrolled 80 obese elderly patients with mild cognitive impairment (MCI) (>60 years old, BMI > 30 kg/m^2^, >80% women, 26.3% APOEε4) and then the intervention group was subjected to CR for 12 months. A total of 75 patients completed the follow-up. The mean BMI decreased by 1.7 ± 1.8 kg/m^2^ (*p* = 0.021), and all groups showed improvement in most cognitive assessments. After adjusting for education, gender, physical activity, and baseline assessments, the decrease in BMI was linked to improvements in verbal memory, verbal fluency, executive function, and global cognition in a study using linear generalized models. For memory and fluency, this connection was strongest among younger seniors, whereas for executive function, it was strongest in APOEε4 carriers. Improvements in cognitive assessments were correlated with changes in the homeostasis model assessment estimates of insulin resistance, C-reactive protein, leptin, energy intake, carbohydrate intake, and fat intake. In summary, in obese adults with MCI, intentional weight loss through CR was safe and associated with improvements in language, global cognition, memory, and executive function; the highest correlation was observed in younger seniors and APOEε4 carriers. Improvements in cognition tests were also linked to dietary modifications and modifications in metabolic indicators [143]. Furthermore, a 2-year intervention conducted in a randomized clinical study enrolling 220 volunteers with a BMI ranging from 22 to 28 kg/m^2^ centered on CR showed significant improvement in working memory [144]. D Given the improvement observed using CR as a dietary intervention, limited clinical reports on IF as the chosen dietary intervention are available. Although limited randomized clinical studies are currently available, there is growing interest in evidence that IF may be associated with improvements in cognitive function [145]. In a randomized controlled dietary intervention trial (NCT02679989), Kim and colleagues investigated the cognitive functions of 43 healthy obese individuals (35–75 years old, WC > 102 cm in men and >88 cm in women, belonging to different ethnic background) subjected to IF or CR for 4 weeks using the mnemonic similarity task approach. This was the first study to compare IF and CR in terms of cognitive function improvement. Patients were randomly assigned to the IF (600 kcal/day for two consecutive days per week) or CR (25% of normal calorie intake) groups. Both diets improved cognitive function in the same way, with no discernible changes. Eating less may improve memory, which is dependent on the hippocampal region, and may be advantageous for older adults whose cognitive abilities are deteriorating. However, beyond the strengths related to the use of a randomized investigation that is the gold standard in human research to evaluate the outcomes of such interventions and the use of the mnemonic similarity task test, some limitations could preclude a correct and definitive analysis. In fact, considering this study, improvement in cognitive function was not the primary outcome of the research; the presence of a higher number of women could constitute a gender-biased analysis, as could the lack of a non-intervention control group [146]. Another interesting randomized controlled trial was conducted by Teong and colleagues comparing the effectiveness of IF and CR in improving cognitive function. They studied the results of 46 healthy women with overweight or obesity (35–70 years old, BMI > 25 kg/m^2^) who completed the 8 weeks of intervention and were randomized into 2 weight loss groups: IF (fasting for 24 h on 3 nonconsecutive days per week; on fasting days breakfast at 08:00 a.m.) and CR (~30% of normal energy intake). The results showed a greater weight loss in the IF group, whereas an increase in cognitive performance was found in both the IF (*p* = 0.036) and CR (*p* = 0.006) groups in one of the cognitive tasks, but there was no statistical difference between groups considering the cognitive functions in this short-term intervention. However, also in this study, there were some limitations: a small group of healthy overweight or obese healthy women, which precludes generalization, and the lack of a noninterventional group [147]. In contrast, Anton and colleagues conducted a pilot study in which 10 sedentary older adults (≥ 65 years old) were subjected to a TRF intervention for 4 weeks (16 h fasting). Adherence to treatment was particularly high (84%). Although a reduction in body weight was observed (−2.6 kg, *p* < 0.01), no significant improvements in secondary outcomes, including the improvement in cognitive function as measured by the Montreal Cognitive Assessment (MoCA), were observed. Clearly, the small sample size is a limitation, as is the absence of a control or comparison group with other dietary regimens [148]. Again, in a prospective cohort clinical study, Ooi and coworkers considered 99 elderly Malaysian males with MCI for the investigation of the effects of different IF approaches on improving cognitive function. At the 36-month follow-up, individuals who frequently practiced IF (regular IF, 2 days fasting per week on Monday and Thursday) showed improved cognitive scores considering several cognitive tests and returned to better cognitive function groups [149]. Finally, two recent systematic reviews reflected the limited and contrasting evidence regarding the use of IF to improve cognitive function. In particular, Senderovich and collaborators conducted the investigation considering nine randomized controlled trials. Notably, some of the included studies indicated that patients with MCI at baseline did not demonstrate any advantage in approaching the mentioned dietary regimen, whereas in some subgroups, extensive lifestyle changes were observed in terms of lessening the intensity of complaints. It was discovered that people with a history of cardiovascular disease may have complained more severely about their inability to solve problems as a result of extensive lifestyle measures [150]. The systematic review conducted by Alkurd and colleagues included 16 clinical studies (all types) in which IF (all types) was used as a dietary intervention, and the outcomes were the evaluation of BDNF levels and cognitive function assessments. They concluded that it is necessary to conduct well-controlled, long-term experimental studies to clarify the effects of CR and IF regimens on BDNF levels and cognitive function because some results are controversial, especially regarding BDNF levels, whereas slight improvements in cognitive function were detected in some subgroups. There was no clear picture of the effects of these dietary interventions on the desired outcome. Consistent with the mentioned study, IF and other fasting diets could provide advantages that were detected that were comparable to those of CR. Accordingly, the current findings further imply that, given its greater applicability and simplicity of use, particularly for older adults and those receiving clinical intervention, IF may be a more effective way to counteract cognitive deterioration associated with aging [151,152]. Briefly, concerning the neurodegenerative disorders Alzheimer’s disease and Parkinson’s disease, in animal models of these diseases, there is compelling preclinical evidence that ADF can postpone the development and advancement of the disease processes [153,154]. By enhancing mitochondrial activity, promoting autophagy, the synthesis of neurotrophic factors, antioxidant defenses, and DNA repair, among other processes, IF could boost the resilience of neurons to stress [154,155]. Furthermore, GABAergic inhibitory neurotransmission, also known as γ-aminobutyric acid-related inhibitory neurotransmission, is improved by IF and can help avoid excitotoxicity and seizures [1]. Accordingly, through its action on ketogenesis, IF may improve cognitive performance. This occurs because the formation of a ketone body functions as an energy source and promotes the survival of neurons under pathological conditions, including ischemia, hypoxia, and anoxia. In addition, it has been demonstrated that ketone bodies have neuroprotective effects by raising ATP levels and lowering the generation of reactive oxygen species (ROS) by increasing nicotinamide adenine dinucleotide (NADH) oxidation and inhibiting mitochondrial permeability transition. In addition, preclinical studies have shown that ketones in Alzheimer’s disease can prevent the pathological form of amyloid-β_1-42_ (Aβ) from entering neurons. Improved synaptic plasticity, decreased oxidative stress, and preserved mitochondrial complex I activity were all brought about by the inhibition of intracellular Aβ accumulation, indicating that IF may have neuroprotective benefits by passing through the ketogenic route and preventing the build-up of Aβ. Furthermore, it was thought that IF could protect against tau protein aggregation and neurofibrillary tangles. The hippocampus, amygdala, and cortex of mice given a precursor of ketone bodies exhibited reduced Aβ and hyperphosphorylated tau deposits [156,157]. These preclinical evidences suggest that providing ketone bodies to patients over an extended period may slow down the progression of the disease and enhance their cognitive abilities. Unfortunately, there are insufficient data from controlled studies on IF in individuals with neurodegenerative disorders or at risk for developing one to provide an accurate conclusion. In fact, there are few human trials examining the impact of IF on dementia and cognitive function, most likely due to issues with trial adherence and sticking to a fasting schedule. Notably, considering the wide-ranging cellular and metabolic effects of IF, it can positively influence neurodegenerative pathogenesis from a variety of perspectives. Providing neuroprotection by improving vascular health and lowering oxidative damage. Other lifestyle therapies, including exercise, on the other hand, have been researched and shown to slow down older people’s cognitive deterioration. The limitations of IF trials may be solved by enrichment studies aimed at dementia-risk populations [158].

According to the results in animals and limited clinical trials, IF can successfully modify a number of risk variables and prevent diseases, and it may modify disease risk to a degree comparable or even better to that of CR. It is important to note that convergence is not evident. In the next subchapter, we describe some interesting and contrasting clinical trials in which IF based on the ADF dietary regimen was employed to investigate different human health benefits related to obese and overweight people and different disorders and related risk factors.

### 2.2. Examples of Clinical Trials Showing ADF-Based Approach as Primary Dietary Intervention

#### 2.2.1. ADF, Weight Loss, and Cardiovascular Health

Obesity increases the individual risk of developing coronary artery disease (CAD), and weight loss remains the gold strategy for improving cardiometabolic health and parameters. ADF as a tool to facilitate weight loss and lower vascular disease risk was thoroughly investigated to assess whether it can produce superior health improvements compared with CR in obese adults. Several short-term studies were performed to establish ADF safety, tolerability, and adherence, as well as to compare changes in weight, body composition, lipids, and insulin sensitivity with those produced by a standard weight-loss diet. In summary, ADF is a safe, efficacious, and tolerable approach to weight loss [81,159] and a viable diet option to help obese individuals lose weight and decrease CAD risk [160] (UIC-004-2009). However, the results for IF and CR in terms of body weight and lipid composition appeared to be similar. In fact, all obese subjects enrolled in these studies showed a decrease in body weight, percentage body fat, total cholesterol, LDL-C, triacylglycerol concentrations, and systolic blood pressure after 8 weeks of diet [159,160,161]. Moreover, the rate of weight loss remained constant during the controlled food intake and self-selected food intake phases [160], and some studies indicated that adherence to ADF was high and did not appear to increase the risk of weight regain 24 weeks after completing the intervention [159]. Similar results were reached when patients were randomized to 1 of 3 groups for 8 weeks according to the fast day mealtime: lunch, dinner, or small meals. No parameters showed significant changes between the three groups, suggesting that there is considerable flexibility in the timing of the fast-day meal during ADF. Obese subjects may consume the meal at dinner or as small meals throughout the day and experience similar weight loss, body composition, and cardiovascular benefits as the traditional lunchtime approach, which has important clinical implications in terms of diet tolerability [162].

Similarly, when ADF studies are performed for the mid-term period, the results suggest that IF is a successful but not superior weight loss approach compared with CR [163,164,165]. Twenty-four obese participants randomized to consume either the 5:2 diet or a standard energy-restricted diet (500 cal reduction per day) for 6 months exhibited a significant reduction in body weight and systolic blood pressure regardless of the dietary intervention. In addition, there was no significant difference in the amount of weight loss or WC reduction, diastolic blood pressure, fasting blood glucose, or blood lipids in either dietary group [164] (ACTRN12614000396628). In contrast, a study conducted on 88 obese women randomly assigned to one of for groups, namely IF70, an IF diet at 70% of calculated baseline energy requirements per week; IF100, an IF diet at 100% of calculated baseline energy requirements per week; CR70, a continuous restriction at 70% of calculated baseline energy requirements daily; or control, 100% of calculated baseline energy requirements daily, for 8 weeks, showed that IF reduced weight and fat mass and improved total and low-density lipoprotein cholesterol more than DR when prescribed at matched energy restriction. However, IF prescribed for energy balance did not improve health compared with other groups, despite modest weight loss [166] (NCT01769976). One hundred overweight and obese participants were randomized to ADF (alternating every 24 h between consuming 25% or 125% of energy needs); CR (consuming 75% of needs every day); or control (consuming 100% of their needs every day) for 24 weeks (NCT00960505). Interestingly, ADF and CR similarly increased the free fat mass:total mass ratio and decreased circulating leptin, without affecting the VAT (visceral adipose tissue):SAT (subcutaneous adipose tissue) ratio or other measured adipokines [163]. These patients were then enrolled in a further 6-month weight-maintenance phase in which participants in the alternate-day fasting group were instructed to consume 50% of their energy needs as a lunch on fast days and 150% of energy needs split between three meals on alternating feast days, while participants in the daily calorie restriction group were instructed to consume 100% of their energy needs split between three meals every day. Although this study bears several limitations related to the short duration of the maintenance phase, the lack of attention received by the control group relative to the intervention groups, the higher dropout rate in the alternate-day fasting group, which may have also introduced a possible selection bias between groups, or the enrollment of metabolically healthy obese individuals, which may have hindered the ability of the interventions to produce greater improvements in measuring cardiovascular disease risk indicators. Results confirmed that the ADF diet was not superior to the daily CR nutritional intervention with regard to adherence, weight loss, weight maintenance, or improvement in risk indicators for cardiovascular disease [12]. Analogous results were obtained by Sundfør and colleagues who investigated the effects of intermittent energy restriction versus continuous energy restriction on weight loss, maintenance, and cardiometabolic risk factors in men and women with abdominal obesity and at least one additional component of metabolic syndrome (NCT02480504). A total of 122 participants were randomized to intermittent or continuous energy restriction. A 6-month weight-loss phase, including 10 visits with dieticians, was followed by a 6-month maintenance phase without additional face-to-face counseling. The intermittent energy restriction group was advised to consume 400/600 kcal (female and male, respectively) on two non-consecutive days. Based on dietary records, both groups reduced energy intake by 26–28% [167]. After 1 year, weight loss was similar among participants in the intermittent and continuous energy restriction groups, and there were favorable improvements in WC, blood pressure, triglycerides, and HDL-C, with no difference between the groups. Weight regain was minimal and similar between the intermittent and continuous energy restriction groups, thus confirming that intermittent energy restriction is as effective, but not superior to continuous energy restriction, at inducing clinically significant weight loss and maintenance and improving cardiometabolic risk factors in free-living men and women with abdominal obesity and at least one additional component of metabolic syndrome [167].

When investigated together with physical exercise to assess the effect on serum sterol signatures, body weight, body composition, and metabolic parameters in overweight or obese adults, evidence suggests that exercise with or without ADF improves cholesterol metabolism and increased physical activity has a greater effect on cholesterol biosynthesis than weight reduction or CR [168]. Of the 112 overweight or obese participants randomly assigned to four groups: ADF and exercise (E-ADF); ADF; exercise; and control, 31 completed the trial. After 8 weeks, the E-ADF and ADF groups lost more body weight and fat mass than the control group; desmosterol, all cholesteryl esters, and oxysterols significantly decreased in the exercise group; and changes in metabolic ratios of desmosterol and 7-DHC to cholesterol, which reflect cholesterol biosynthesis, negatively correlated with changes in physical activity but not with changes in calorie intake or body weight [168].

#### 2.2.2. ADF and Metabolic Health

The benefits of a very low-calorie diet on glucose homeostasis are well recognized, and IF has popular appeal, although implementation in people with diabetes can be discouraging in view of the risk of hypoglycemia caused by changing the requirements for medication with weight loss. Nonetheless, experimental and preliminary clinical data indicate that fasting may not only reduce body weight but also improve insulin sensitivity and have beneficial effects on blood pressure. Li and colleagues investigated the effects of a 1-week fasting period compared with usual care in T2DM. Forty-six diabetic patients were enrolled in the study and were divided into fasting or control groups; of the fasting group, only 17 participants out of 23 completed the study. The fasting program consisted of 2 pre-fasting days where subjects received a low-calorie (approx. 1200 kcal) and low-salt diet with an intake of pure cooked rice and vegetables only, followed by 7 modified fasting days where participants received unrestricted amounts of water, herbal tea (no black or green tea), 200 mL fruit juice, and small standardized quantities of light vegetable soup with a maximum total daily energy intake of 300 kcal. A norm caloric diet was reached again thereafter, and the participants were then advised to follow the recommendations of a Mediterranean diet. People allocated to the control group were advised to follow the principles of a Mediterranean diet. Fasting took place only once in the 4-month period, and outcomes were assessed at baseline and after 4 months. Despite limitations, namely the small size of the population in a single center, the group differences between the control and fasting set that may introduce bias in the group comparisons, the absence of outcome assessment immediately after the fasting intervention, and the lack of a detailed dietary adherence assessment, the results of this study suggested that fasting was well accepted without any serious adverse events. After 4 months, the mean weight decreased by 3.5 kg and 2.0 kg in the fasting vs. control group, respectively, consistent with a greater reduction in abdominal circumference. Fasting led to a significant decrease in systolic/diastolic blood pressure and increased quality of life, whereas for HbA1c, insulin, and HOMA-index, only non-significant improvements were observed [169]. In a non-blinded randomized parallel group interventional trial, participants with T2DM treated with metformin and/or hypoglycemic medication, followed a 500–600 kcal diet for 2 days per week for 12 weeks. A total of 41 participants were randomized 1:1 to consecutive (*n* = 19) or non-consecutive (*n* = 22) day fasts to establish whether the risk of hypoglycemia was greater with 2 consecutive days of a very-low-calorie diet than with 2 non-consecutive days of a very-low-calorie diet. Evidence shows that IF was associated with a twofold increase in hypoglycemia on fasting days; however, there were no episodes of severe hypoglycemia, and most participants did not experience hypoglycemia. Because of the low overall hypoglycemia event rate, it was not possible to determine if there was a significant difference in hypoglycemia between the treatment arms. These observations suggest that the risk of hypoglycemia appears to be more dependent on individual characteristics than on the fasting pattern. Moreover, the intervention resulted in weight loss, reduced HbA1c levels, and a small improvement in the quality of life experienced by patients in both arms [170]. Interestingly, when compared with daily CR, ADF produces superior reductions in HOMA-IR (a marker of insulin resistance).

Forty-three insulin-resistance participants who underwent a 12-month study that compared ADF (25% energy needs on fast days; 125% energy needs on alternating feast days) with CR (75% energy needs every day) were further examined to assess the effects of alternate-day fasting with those of daily CR on body weight and glucoregulatory factors. In insulin-resistant participants, weight loss was not different between ADF and CR by month 12, relative to controls, and fat mass and BMI decreased similarly in ADF and CR. Nevertheless, ADF produced greater decreases in fasting insulin and insulin resistance compared with CR and the control regimen by month 12 [171].

Insulin resistance, abdominal obesity, hyperglycemia, hypertension, and dyslipidemia define a metabolic disorder known as MetS, which is a major and prevalent risk factor for cardiovascular disease and diabetes. In a single-center, randomized clinical trial (IRCT201509092395N8). Arefe and colleagues compared the effects of calorie restriction and a modified alternate-day fasting diet on the treatment of adults with metabolic syndrome. Seventy participants diagnosed with metabolic syndrome were randomly allocated into two groups to follow either calorie restriction or a modified alternate-day fasting diet for 8 weeks (75% energy restriction during the 3 fast days and then ate a diet that providing 100% of their energy needs on each feed day). Anthropometric parameters, blood pressure, fasting plasma glucose, fasting insulin, HOMA-IR, and lipid profile were measured at baseline and after the conclusion of the trial. The results demonstrated that compared with a CR diet, adherence to an ADF diet has a more beneficial effect on reducing body weight and WC, improving systolic blood pressure and fasting plasma glucose levels. However, these findings do not suggest any difference between the ADF and CR diets in terms of BMI, lipid profile, or diastolic blood pressure. Although a greater reduction in fasting insulin levels and HOMA-IR were detected in the ADF group, these changes did not reach statistical significance when compared with CR [172]. Patients with MetS were also analyzed to examine the effects of IF on cardiometabolic health and gut microbiota. Intervention consisted of 8 weeks of “2-day” modified IF, in which 39 patients (*n* = 21 in the IF group and *n* = 18 in the control group) were included. In the following randomized clinical trial (NCT03608800), patients in the IF group reduced their daily energy intake by 75% for 2 nonconsecutive days a week and followed an ad libitum diet the other 5 days, for 8 weeks. The 8-week IF caused significant changes in circulating biomarkers, including those for inflammation, oxidative stress, and endothelial function, thus resulting in systemic anti-inflammatory effects, as evidenced by significantly decreased circulating levels of sCD40L, which is known to play an essential role in platelet activation and atherogenesis. Gut-related metabolites, including LPS and SCFAs, also improved. Importantly, these effects appear to be associated with alterations in gut microbiota composition, microbial-related metabolites, and activated metabolic pathways in the gut microbiome. IF resulted in gut bacteria alteration and activated microbial metabolic pathways that were strongly associated with improvements in cardiovascular biomarkers. Similar to other IF studies, a 4.0% reduction in body weight was observed, which was paralleled by a significant decrease in fat mass and visceral fat. Significant improvements were also observed in serum triglycerides, insulin, and HOMA-IR within the IF group although the was not a significant effect of 8-week 2-day IF on dyslipidemia and glucose metabolism compared with the control group [173]. A recent network meta-analysis conducted by Xiaoyu and colleagues confirmed the potential beneficial effects of ADF on T2DM patients. In 13 considered randomized controlled trials (*n* = 867) in which several IF interventions were analyzed. Among them, the modified ADF approach, the IF 5:2 diet, was more effective in improving fasting blood glucose, HbA1c, and insulin resistance, although some studies did not show significant differences among various IF regimens [174].

It is noteworthy that there is still debate about the safety and efficiency of CR and IF, particularly in healthy humans. In fact, although chronically increased caloric intake has negative effects on human health and CR is known to extend health span and lifespan in model organisms, continuous CR has also been associated with depleted circulating leukocytes, immunosuppression, and reduced bone density [175,176,177]. Moreover, some studies have shown the adverse effects of recurring fasting periods, as discussed in some cohorts where skipping breakfast is associated with an elevated risk of coronary heart disease, T2DM, and other adverse factors [178,179]. Stekovic and colleagues examined the effects of strict ADF on cardiovascular parameters, such as heart rate, blood pressure, cholesterol levels, cardiovascular disease risk, body composition, and the metabolome and proteome of healthy, non-overweight adults, in order to assess both the effectiveness and safety of such intervention [180]. Thirty long-term ADF healthy adults who had been performing ADF for more than 6 months on their own prior to enrollment in the study were compared to 60 healthy, non-ADF performing controls in a cross-sectional analysis followed by a randomized controlled trial where all subjects of the control group were further randomized to either control (which continued their current ad libitum eating behaviors) or 4-week ADF intervention group in a 1:1 ratio (NCT02673515). What emerges from these studies is that individuals performing ADF in the randomized controlled trial did not fully compensate for the lack of caloric intake on the fasting days with elevated calorie intake on the feast days, thus reaching significant levels of CR (37.4%) throughout the intervention, which led to a reduction in BMI by more than 1 kg/m^2^. Analyses of body composition by dual-energy X-ray absorptiometry (DEXA) revealed that fat reduction preferably influenced the trunk fat, in particular the android area, which is believed to be the most lipotoxic one [181,182]. Despite a statistically significant body weight reduction in the ADF vs. control group in the 4-week intervention trial, no changes in insulin sensitivity were observed. It seems plausible to assume that in healthy individuals who are already highly insulin sensitive at baseline, ADF does not further improve the parameters of insulin sensitivity. Similarly, no clinically or statistically relevant differences were detected in insulin sensitivity parameters between the long-term ADF and control groups. According to these data, ADF may lead to improved cardiovascular health because it significantly reduced the Framingham risk score (risk in percent to develop a cardiovascular disease in the next 10 years) after 4 weeks, showing improved cardiovascular markers, reduced systolic and diastolic blood pressure, heart rate, arterial and pulse pressure, and pulse wave velocity, while having a significant impact on blood lipids after >6 months of intervention. On the other hand, >6 months of ADF did not cause a decline in bone mineral density or white blood cell count, as reported for longer periods of constant CR [177,183,184], which makes this nutritional intervention a suitable alternative to continuous CR. To support the contribution of ADF to long-term health span improvements and cardioprotective effects, analyses of the metabolic and proteomic changes that occur between fast and feast days revealed that pathways of essential PUFAs omega-3/6 linolenic and linoleic, respectively, and arachidonic acid concomitantly with long chain fatty acids β-oxidation were enriched, whereas metabolites from the urea cycle, ammonia recycling, and several pathways associated with pro-aging amino acid metabolism were substantially depleted. Furthermore, serum levels of ketone bodies such as BHB, which is related to anti-aging and cardioprotective properties [185], were found to be still elevated after >6 months of ADF on fasting days; interestingly, after 4-weeks intervention BHB levels were high even on samplings collected on non-fasting days, suggesting persistently changed ketone metabolism due to the rhythmic fasting periods. The study also revealed the modulation of the thyroid axis by the periodic depletion of energy intake: short-term ADF was sufficient to reduce circulating triiodothyronine (fT3) levels, which is maintained and consistent with a greater secretion of para-thyroid hormone (PTH) in those practicing ADF for >6 months. Because there was no difference in the circulating levels of thyroid-stimulating hormone (TSH) and free thyroxine, providing evidence for normal function of the thyroid gland, low levels of fT3 have been intensively linked to longevity in humans [186]. Although there are several limitations due to the lack of baseline values for the long-term cohort, the relatively low number of participants, or the approach to the recruitment of subjects that could have introduced a selection bias toward participants who were already knowledgeable and/or interested in ADF, this study sheds light on the physiological impact of ADF and eventually supports its safety as a clinically relevant intervention [180]. The ADF studies included in this narrative review are detailed in Table 3.

### 2.3. Concluding Remarks on IF: Limitations, Undesired Effects, Changes in Blood Profile and Anthropometric Parameters

In summary, some observations are worth considering:

- This narrative review only considered publications, reports, and clinical trials published in English. It is well known that there are many materials on IF in other languages (i.e., Chinese and Hindi). This represents a possible bias in the analysis.
- All of the reviewed studies have several limitations. First, the small sample sizes, including a maximum of approximately 100 participants, question whether these studies could adequately detect statistically significant differences in primary and secondary outcome measures; secondly, these studies mainly analyzed overweight adults living in one geographical region, namely the USA (it is not surprisingly considering that in the USA, obesity is reaching epidemic proportion), making it difficult to generalize to other age groups, populations, and food cultures to tailor recommendations and personalize the approach, although we also considered studies from other countries and systematic reviews and meta-analyses that considered various populations. Third, the duration of the studies is often limited, and if, on the one hand, it may impede the detection of significant changes in some parameters, on the other hand, data on feasibility and long-term efficacy are lacking. Moreover, blinding study personnel or participants is not always possible because of the nature of the dietary interventions. Lastly, the introduction of accidental bias during the recruitment phase, rather than missing participant outcome data or self-reporting records, may have resulted in misstatements [1,84,172,180].
- The blood lipid profile is usually positively influenced by IF and CR, suggesting that these two interventions have equivalent effects on these lipid parameters. However, levels of HDL-C, by contrast, generally remain unchanged or slightly decrease with IF [159,162,165]. In fact, HDL-C is rarely improved by changing dietary patterns alone, but it is generally augmented as an answer to endurance exercise training [84,160,187,188,189,190]. The modification of blood lipid profile is strictly related to the degree of weight loss achieved, which is usually similar between IF and CR, although ADF results in a greater relative reduction in fat mass and provides relative preservation of fat-free mass [88]. Since WC is an indicator of visceral fat mass, its greater reduction appears consistent with results attained by ADF intervention [172].
- IF may benefit several different populations, including those with obesity and overweight [159,160,162,163,164,165,167,168], insulin resistance, prediabetes, T2DM [169,170,171], MetS [172,173], and type I diabetes [191]. Furthermore, the efficacy of IF in reducing body weight does not appear to vary according to an individual’s sex or menopausal status [166,192]. Although research is still in its infancy, it is possible that IF may decrease androgen markers in both genders, resulting in a valuable tool for treating hyperandrogenism in females with polycystic ovary syndrome (PCOS) by improving menstruation, fertility, and quality of life. However, IF could be a less desirable option among males because low testosterone levels can negatively affect metabolic health, muscle mass synthesis, and libido [193]. IF is not recommended in individuals with hormonal imbalances; pregnant and breastfeeding women or young children, as no study to date has evaluated the safety of these diets in these population groups; adults of advanced age, as the effects of fasting on aging-induced sarcopenia remain unclear and they have a higher risk of dehydration and malnutrition; and individuals with immune deficiencies [4,73,84]. Despite IF being regarded as generally safe, diabetic patients treated with sulfonylureas or insulin should carefully consider whether IF is the most suitable option and should not undertake this procedure without the help of their physician because of the risk of hypoglycemia. Both dose adjustment and medication discontinuation should be carefully evaluated during ADF according to fasting blood glucose levels [73,84]. Medical supervision is required, especially for adolescents with obesity, for whom IF might be effective for weight control when weight loss is clinically indicated. In particular, as adolescence is the life stage with the highest rate of eating disorder development, the choice between IF and CR should be deeply considered. IF is not a suitable intervention for individuals with a history of eating disorders [84].
- Dehydration, nausea, headache, dizziness, syncope, weakness, cold, and irritable feelings; bad breath, low energy, and hunger pangs represent the most common undesirable effects of IF. These conditions are usually mild and do not hinder participants from continuing their fasting. Many side effects are linked to dehydration; therefore, it is advisable to consume at least 1.5 L of water per day; vitamin and/or mineral supplements, together with 50 g of lean protein consumption on the fast day of IF regimens, help to control hunger, prevent excessive lean mass loss, and control feelings of weakness [73,84].
- Adherence to IF regimens could be superior with respect to CR intervention; however, it usually decreases when the duration of intervention reaches 12 weeks or longer [194]. Furthermore, most physicians are not trained to prescribe specific IF interventions, and they can hardly follow their patients through their journey [1,4,73].
- The effect of diet on mood may also influence adherence: some trials showed that IF had a positive effect on mood over CR, with bad temper being reported only in 3% of the participants; however, in a trial with lean participants, an overall worsening of mood was reported [195]. As a consequence, fasting can be challenging, and hence its practicality in the modern lifestyle can sometimes be debatable. Nevertheless, IF interventions could be easily tailored to patients’ needs without hindering their results in terms of metabolic health and weight loss. Interestingly, exercise during ADF appears to attenuate the dropout rate [188,196,197].

## 3. Cellular Pathways Influence by Food-Intake Restriction

Each IF intervention can provoke a series of physiological responses by modifying numerous cellular pathways, exerting potential beneficial effects on the human body. In particular, all IF interventions are involved in a process called “metabolic switch”, occurring when IF is maintained for a sufficient period. Depending on the beginning hepatic glycogen content, the make-up of the last meal, and the quantity of energy expended by the individual throughout the fast, this process takes place from 12 to 36 h after the fasting period starts [3,152,198]. Turning on the metabolic switch means that the body will no longer prefer to obtain energy from glucose, but rather from lipids (triglycerides) in adipose tissue that has been stored as fat. Following their release, lipids are broken down into free fatty acids and are first converted to the intermediate stage of acetyl CoA by the β-oxidation process, which also produces the ketones acetoacetate and BHB in the liver [153]. Numerous proteins and biomolecules that are known to affect aging and health are regulated by ketone bodies in terms of their expression and function [1]. The most important include FGF-21, poly (ADP-ribose) polymerase 1 (PARP1), ADP ribosyl cyclase (CD38), Fork head box Os (FOXOs), NF-κB, sirtuins (SIRTs), and peroxisome proliferator–activated receptor γ coactivator 1α (PGC-1α). It is well known that cellular stress, fasting, or intense exercise can activate SIRT1, and by deacetylating PGC-1α activates nuclear respiratory factor 1 (NRF-1), which controls the expression of genes involved in metabolic response, mitochondrial respiration, and mitochondrial DNA transcription and replication. However, as a coactivator protein, PGC-1α reacts to external stimuli and then modulates many pathways in a highly coordinated, tissue-specific way [199,200]. For example, in cardiomyocytes the activation of SIRT1, leading to the activation of PGC-1α, was related to cardioprotective effects [201,202,203]. Furthermore, PGC-1α can regulate the transcription of NRF2, leading to the activation of the KEAP1/NRF2 pathway, culminating in the transcription of several antioxidant and cytoprotective genes (i.e., superoxide dismutase (SOD), heme oxygenase 1 (HO-1), catalase, etc.) [204]. IF can stimulate autophagic processes, leading to the removal of oxidatively injured cells [1]. In fact, from the blood, acetoacetate and BHB are carried to the brain and subsequently into the neurons. Apart from the metabolites of ketones in the liver, astrocytes can also undergo ketogenesis, potentially serving as a significant local source of BHB for neurons. Lowering the AMP:ATP ratio in neurons due to decreased glucose availability and increased ketones triggers the kinases AMPK and CaKMII, which in turn trigger autophagy through PGC-1α and CREB activation. Moreover, the mTOR pathway becomes less active during fasting because of decreased glucose levels, which trigger autophagy [205]. Autophagy is also in charge of the body’s defense against oxidative stress, which involves the build-up of dangerous free radicals and is a condition that worsens with age and the progression of neurodegenerative disorders [155]. Increased antioxidant defenses (molecules that stop free radicals from oxidizing), DNA repair, and BDNF stimulation are brought about by the inhibition of the mTOR system. Additionally, BHB has the ability to increase the expression of BDNF, which may support cellular stress tolerance, synaptic plasticity, and mitochondrial biogenesis. Because IF decreases the blood level of circulating insulin, the insulin/IGF-1 signaling (IIS) pathway benefits neuroplasticity and provides defense against oxidative and metabolic stress [3]. By increasing insulin sensitivity, intermittent fasting may potentially have a positive indirect impact on the brain. Patients with diabetes have reduced insulin sensitivities, which affect how well glucose is absorbed by cells; however, this deterioration naturally occurs as people age [206]. Because IF lowers blood levels of circulating insulin, insulin receptor sensitivity is increased, and the IIS pathway is upregulated, which improves neuronal uptake and glucose utilization. Increased cellular plasticity and defense against oxidative stress are linked to upregulation of IIS activity, which also lowers the activity of the mTOR pathway [153].

**Figure 2:**
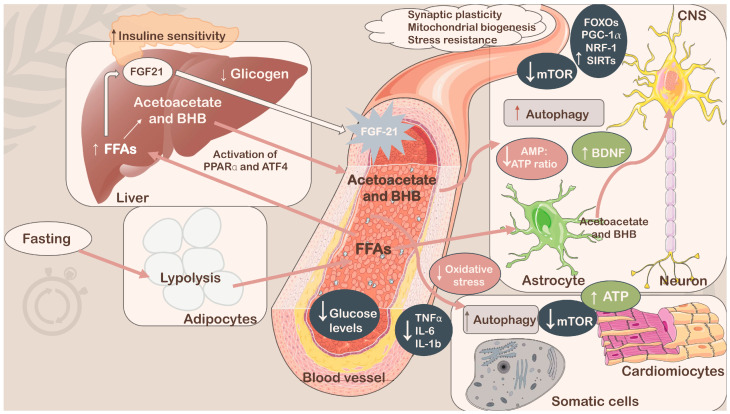
Schematic representation of the main cellular mechanism modified by fasting adapted from [1,152]. Abbreviation: β-hydroxybutyrate (BHB), free fatty acids (FFAs), brain-derived neurotrophic factor (BDNF), central nervous system (CNS), brain-derived neurotrophic factor (BDNF), tumor necrosis factor alpha (TNFα), IL (interleukin), fibroblast growth factor 21 (FGF-21). The picture was prepared by using Smart SERVIER MEDICAL ART provided under Creative Commons Attribution 3.0 Unported License.

Regarding IF and inflammation, it has been demonstrated that diets high in glucose may cause the synthesis of proinflammatory factors (TNFα, IL-6, and IL-1b), and IF, by reducing these proinflammatory molecules, may help to lower oxidative stress and systemic inflammation, both of which contribute to the development of atherosclerosis [98]. Figure 2 illustrates the main changes in IF-induced cellular pathways.

## 4. Conclusions and Future Perspectives

In this review article, we analyzed dietary interventions based on IF, which are mainly used to lower body weight, reduce risk factors for disorders, and improve health benefits. Regrettably, it is necessary to improve the quality and quantity of clinical trials to provide evidence to put an end to this nutritional intervention. In fact, if from one side some investigations showed significant results on the proposed outcomes, other ones were found in contrast with the positive findings. Furthermore, it is not possible to establish a superior profile of IF with respect to CR. Contradictions should be quickly resolved to restrict skepticism and limit overemphasis on this nutritional approach. Furthermore, considering that to reduce weight and strengthen heart health, cutting back on daily eating time to a maximum of 8 h a day has become increasingly common in recent years, we need to have a clear perception of the long-term health implications of time restrictions on eating, such as disease risk factors. Moreover, considering the extremely recent issues on death related to a specific TRF approach raised in a new report, before sponsoring and proposing IF as a gold standard for body weight management, further and more convincing evidence is required. Although promising, the IF dietary regimen has not yet demonstrated a clearly superior profile compared with a well-established nutritional intervention based on CR. It is expected that future clinical studies will shed light on the darkness that currently exists around IF.

## Figures and Tables

**Table 2:** Schematic view of advantages and disadvantages of different IF approaches.

IF Method	Advantages	Disadvantages
ADF Alternate day fasting	Easy to implement in the existent dietary regimen Compatible and sustainable with the patient lifestyle Few rules easy to follow Eating what and how the patient wants without calorie counting in non-fasting day	Not indicated to some sub-population: children, pre-teens, person with eating disorders, pregnant/lactating women, people with disorders (liver disorders (i.e., Gilbert syndrome), hypoglycemia, type I diabetes, etc.) and nutrient deficits Influence on social life At the beginning difficulties due to hunger, headache, constipation, and fatigue Encourage binge eating disorders
IF 5:2	Rotate between periods of fasting and eating Do not have to diet all week long Eating what and how the patient wants without calorie counting in non-fasting days Possibility to choose days to fast on, and days to eat	Not indicated to some sub-population: children, pre-teens, person with eating disorders, pregnant/lactating women, people with disorders (liver disorders (i.e., Gilbert syndrome), hypoglycemia, Type I diabetes, etc.) and nutrient deficits Junk food you should not eat during eating 5 days Not suitable for people who are not able to fast Experienced undesired effects, such as hunger pains, sleeplessness, headache, and irritability on fasting days It is not particularly structured Risk of overeating
FMD Fasting-mimicking diet	Structured dietary regimen (small amounts of food are consumed to mimic the effects of fasting), with specific amounts of macro- and micronutrient (43–47% carbohydrate; 44–46% fat; 9 to 11% protein) FMD facilitates weight loss Sustainable weight management FMD could improve motor coordination and memory Easy to implement in the existent dietary regimen through cycles. Two to five consecutive days fasting, and the return to a normal diet for the rest of the month	Not indicated to some sub-population: children, pre-teens, person with eating disorders, pregnant/lactating women, people with disorders (liver disorders (i.e., Gilbert syndrome), hypoglycemia, Type I diabetes, etc.) and nutrient deficits Due to the prolonged complete fasting (consuming only water for several days in PF) or very-low-calorie diets, it is not suitable for everyone Experienced undesired effects, such as fatigue, weakness, and headache Limited data on its long-term safety, efficacy, and sustainability Risk of malnutrition and overeating issues after fasting
PF Periodic fasting		
TRF Time-restricting feeding	Simple and flexible: no calorie restriction like other forms of fasting–eating until the patient is satisfied each day, as long as the patient wait the specific hours before eating again	Not indicated to some sub-population: children, pre-teens, person with eating disorders, pregnant/lactating women, people with disorders (liver disorders (i.e., Gilbert syndrome), hypoglycemia, Type I diabetes, etc.) and nutrient deficits Experienced undesired effects, such as extreme hunger, fatigue, dizziness, weakness, fainting, fast heartbeat, heart palpitations, and headache Not suitable for anyone, could be a source of stress, especially in individual with compromised or high cortisol and adrenaline levels
eTRF Early time-restricting feeding	eTRF improves circadian misalignments, and eating in accordance with daylight signals promotes better health	
lTRF Late time-restricting feeding	lTRF is a good option for people who prefer to socialize or eat dinner later in the day	

**Table 3:** Overview of the selected clinical studies based on ADF approach.

	Clinical Trial	Participants	Trials Weeks	Intervention	Body Weight (kg)	WC	Blood Pressure (mmHg)	Plasma Lipids (mg/dL)	Glucoregulatory Factors (mg/dL)	Adherence and Tolerability	Ref.
**Overweight and obese**	UIC-004-2009	16 obese subjects (12 females, 4 males); age 35–65 y, BMI 30–39.9 kg/m^2^	10	3 intervention phases: 2-w pre-loss control phase (usual eating and exercise habits) 4-w weight loss/ADF controlled food intake phase (25% of energy needs on the fast day (24 h) and ad libitum food on each alternate feed day (24 h)) 4-w weight loss/ADF self-selected food intake phase (25% of energy needs on the fast day and ad libitum food on the feed day)	−5.8	NA	SBP −9.5	TG −38	FI NA	No drop in adherence during the different phases	[160]
								TC −37			
									FPG NA		
							DBP −1.5				
								LDL-C −29			
									HOMA-IR NA		
								HDL-C +2			
	Randomized, parallel-arm feeding trial	74 subjects with BMI 30–39.9 kg/m^2^; age 25–65 y	10	ADF lunch (ADF-L): 25% of baseline energy needs on the fast day (24 h), and ad libitum on each alternating feed day (24 h)	−3.5	NA	SBP −2	TG −6	FI 0 μIU/mL	Adherence to the fast day energy goal was similar for each group. Compliance of ADF meal was high in each intervention group	[162]
								TC −1			
									FPG −2		
							DBP −1	LDL-C −2			
									HOMA-IR −0.3		
								HDL-C −2			
				ADF dinner (ADF-D): 25% of baseline energy needs on the fast day (24 h), and ad libitum on each alternating feed day (24 h)	−4.1	NA	SBP −5	TG −9	FI −2 μIU/mL		
								TC −5			
									FPG −1		
							DBP −3	LDL-C 0			
									HOMA-IR −0.8		
								HDL-C 0			
				ADF-small meals (ADF-SM): 25% of baseline energy needs on the fast day (24 h), and ad libitum on each alternating feed day (24 h)	−4	NA	SBP −6	TG −1	FI −2 μIU/mL		
								TC −1			
									FPG −2		
							DBP −1	LDL-C +1			
									HOMA-IR −0.8		
								HDL-C −1			
	Pilot study	26 subjects, aged 18–55 y; BMI 30 kg/m^2^	8	zero-calorie ADF (*n* = 14)	−8.2	NA	SBP NA	TG −25	FI +3 μU/mL FPG +6 HOMA-IR NA	No relevant safety changes over the 8-w; zero-cal ADF is safe and tolerable and not associated with weight regain after 24 w of follow-up	[159]
								TC −31.8			
							DBP NA	LDL-C −22.6			
								HDL-C −4.2			
				CR (*n* = 12): −400 kcal/day	−7.1	NA	SBP NA	TG −2.8	FI −0.2 μU/mL FPG +3.3 HOMA-IR NA		
								TC −21.7			
							DBP NA	LDL-C −16.9			
								HDL-C −4.2			
	Randomized controlled trial NCT00960505	79 subjects, BMI 25–40 kg/m^2^; men and women aged 18–65 y	24 (12 control feeding period, 12 self-selected feeding)	ADF (*n* = 25) alternating every 24 h between consuming 25% or 125% of energy needs	NA	NA	SBP NA	TG NA	FI −7.4 μIU/mL	NA	[163]
								TC NA			
									FPG 0		
							DBP NA	LDL-C NA			
									HOMA-IR −1.88		
								HDL-C NA			
				CR (*n* = 29) consuming 75% of needs every day	NA	NA	SBP NA	TG NA	FI −4.4 μIU/mL		
								TC NA			
									FPG +5.2		
							DBP NA	LDL-C NA			
									HOMA-IR −0.79		
								HDL-C NA			
				CONTROL (*n* = 25) consuming 100% of needs every day	NA	NA	SBP NA	TG NA	FI +0.6 μIU/mL		
								TC NA			
									FPG +5.2		
							DBP NA	LDL-C NA			
									HOMA-IR +0.5		
								HDL-C NA			
	Randomized, controlled, long-term study NCT02480504	112 subjects, aged 21–70 y; BMI 30–45 kg/m^2^	1 y (6 months weight loss, 6 months weight maintenance)	5:2 approach consumption of 400/600 kcal (female/male) on each of two nonconsecutive days a week and to consume food as usual the remaining five days a week	−9.1	−8	SBP −1.9	TG −0.31 mmol/L	FI NA	None of the participants withdrew. Participants in the IF group reported stronger feelings of hunger. Adverse events and larger weight regain than in the CR group	[167]
								TC +0.7 mmol/L			
									FPG −0.2 mmol/L		
							DBP −3				
								LDL-C −0.03 mmol/L			
									HOMA-IR NA		
								HDL-C +0.13 mmol/L			
				CR reduction in energy intake seven days a week	−9.4	−9.2	SBP −3.6	TG −0.11 mmol/L	FI NA		
								TC +0.17 mmol/L			
									FPG 0 mmol/L		
							DBP −2.9				
								LDL-C +0.08 mmol/L			
									HOMA-IR NA		
								HDL-C +0.13 mmol/L			
	Single-center, parallel group randomized controlled trial ACTRN12614000396628	23 males, aged 55–75 y, BMI ≥ 30 kg/m^2^	6 months	5:2 approach *n* = 11 restricted daily calorie intake to 600 cal on the fast day for two non-consecutive days per week and eat ad libitum on the remaining 5 days	−5.3	−8	SBP −14	TG −0.3 mmol/L	FI NA	No adverse side effects experienced in either dietary group. Over half of participants on the 5:2 diet experienced hunger after 2 w with slight progress over time- Compliance rates were similar in both dietary groups	[164]
								TC 0 mmol/L			
									FPG −0.1 mmol/L		
							DBP −0.2	LDL-C −0.09 mml/L			
									HOMA-IR NA		
								HDL-C +0.04 mmol/L			
				SERD (standard energy-reduced diet) *n* = 12 continuous daily energy-restricted diet (500- cal daily reduction from average requirement)	−5.5	−6.4	SBP −10.2	TG −0.2 mmol/L	FI NA		
								TC +0.2 mmol/L			
									FPG −0.2 mmol/L		
							DBP −3.7	LDL-C −0.45 mmol/L			
									HOMA-IR NA		
								HDL-C 0 mmol/L			
	Randomized, controlled, parallel-arm diet trial NCT0365253	31 subjects, aged 20–65 y; BMI >23 kg/m^2^	8	E-ADF (ADF and exercise) 25% of daily recommended energy intake (approximately 500 kcal) on each fast day (24 h), and food ad libitum on each feed day (24 h). The fast day and feed day were repeated every other day, and the fast day occurred 3 days per week. Exercise-training and aerobic exercise.	−3.9	NA	SBP NA	TG −43.6	FI −3.87 μIU/mL	NA	[168]
								TC +15.1			
									FPG −14.1		
							DBP NA	LDL-C +17.8			
									HOMA-IR −1.12		
								HDL-C +6			
				ADF 25% of daily energy intake (approximately 500 kcal) on each fast day (24 h), and food ad libitum on each feed day (24 h). The fast day and feed day were repeated every other day, and the fast day occurred 3 days per week	−3.9	NA	SBP NA	TG +12.6	FI +3.21 μIU/mL		
								TC +5.4			
									FPG −9.7		
							DBP NA				
								LDL-C 0			
									HOMA-IR +0.68		
								HDL-C +2.9			
				EXERCISE exercise intervention included resistance training and aerobic exercise.	−2	NA	SBP NA	TG −87.9l	FI +0.04 μIU/mL		
								TC +20.3			
									FPG −1.3		
							DBP NA	LDL-C +26.7			
									HOMA-IR +0.01		
								HDL-C +11.2			
				CONTROL	−0.2	NA	SBP NA	TG +53.2	FI +5.19 μIU/mL		
								TC +33.2			
									FPG −4		
							DBP NA	LDL-C +16.9			
									HOMA-IR +1.10		
								HDL-C +5.7			
	Longitudinal study	31 subjects, BMI 30–49.9 kg/m^2^; aged 18–65 y	6 months (3 months weight-loss, 3 months weight-maintenance)	ADF low-carbohydrate intervention	−5.5	NA	SBP −7	TG −14	FI −4 μIU/mL	Adherence was high amongst those who completed the study. High dropout (40%), particularly in the first few months of intervention	[165]
								TC −12			
									FPG 0		
							DBP −4	LDL-C −10l			
									HOMA-IR −0.7		
								HDL-C −2			
**T2DM**	Non-blinded randomized parallel group interventional trial ACTRN12614000402640	37 subjects aged >18 y with T2DM treated with metformin and/or any combination of hypoglycemic agents, HbA1c 50–86 mmol/mol; 15 females and 22 males.	12	Diet with consecutive fasting days (*n* = 18)	−3.1	−1.6	SBP −3	TG +0.1 mmol/dL	FI NA	Hypoglycemic events (53) during 84 days of observation affecting 15 participants which required further medication adjustments in 9 out of 37 subjects	[170]
								TC +0.1 mmol/dL			
									FPG −1.3 mmol/dL		
							DBP −2	LDL-C +0.15 mmol/dL			
									HOMA-IR NA		
								HDL-C +0.1 mmol/dL			
				Diet with non-consecutive fasting days (*n* = 19)	−3.6	−3.4	SBP −4	TG 0.1 mmol/dL	FI NA		
								TC −0.4 mmol/dL			
									FPG −1.1 mmol/dL		
							DBP −3				
								LDL-C −0.1 mmol/dL			
									HOMA-IR NA		
								HDL-C 0 mmol/dL			
	NCT00960505	43 insulin-resistant individuals, aged 18–65 y, BMI 25–39.9 kg/m^2^	12 months	ADF (*n* = 11) 6 months weight-loss: 25% of baseline energy needs as a lunch on fast days and 125% of baseline energy needs over three meals on alternating feast days 6 months maintenance phase: 50% of energy needs as a lunch on fast days and 150% of energy needs over three meals on alternating feast days	−8	NA	SBP −9	TG −27	FI −12 μIU/mL	ADF participants consumed almost twice as many calories on fast days but still observed greater metabolic effects compared with CR participants	[171]
								TC +4			
									FPG −3		
							DBP −5	LDL-C +7			
									HOMA-IR −3		
								HDL-C +3			
				CR (*n* = 17) 6 months weight-loss: 75% of baseline energy needs over three meals every day 6 months maintenance phase: 100% of energy needs over three meals every day	−5	NA	SBP −7 mmHg	TG −6	FI −1 μIU/mL		
								TC −6			
									FPG −4		
							DBP −2	LDL-C −6			
									HOMA-IR −0.9		
								HDL-C +2			
				CONTROL (*n* = 15) not changing usual eating and activity habits	0	NA	SBP −1	TG −8	FI −3 μIU/mL		
								TC −1			
									FPG +4		
							DBP −3	LDL-C 0			
									HOMA-IR 0.5		
								HDL-C +2			
	Randomized controlled clinical pilot study	32 subjects, aged 25–75 y with a manifest and treated T2DM	7 days of intervention 4 months trial	ADF *n* = 16 2 pre-fasting days with moderate caloric restriction (approx. 1200 kcal) followed by 7 modified fasting days with nutritional energy intake of 300 kcal/day by liquids only and subsequent stepwise re-introduction of ordinary food items over 3 days	−3.5	−4.4	SBP −13.9	TG −26.6	FI −3.5 μU/mL	Fasting was well accepted, no serious adverse events	[169]
								TC −0.5			
									FPG −10.6		
							DBP −9	LDL-C −2.6 l			
									HOMA-IR −1.5		
								HDL-C +6.5			
				CONTROL *n* = 16 following the principles of a Mediterranean diet	−2	−0.3	SPB +0.4	TG −2.5	FI −0.2 μU/mL		
								TC −15.5			
									FPG −38.4		
							DBP +3.2	LDL-C −7.8			
									HOMA-IR −1.5		
								HDL-C −2.3			
**Metabolic syndrome**	Single-center, randomized clinical trial IRCT201509092395N8	69 subjects, aged 25–60 y overweight (BMI 25–40 kg/m^2^), 41 males and 28 females diagnosed with MetS	8	ADF *n* = 35 75% energy restriction during 3 fast days and 100% of energy needs on feed day	−4.1	−4	SBP −13	TG −52	FI −2.41 μU/mL	no complaint due to difficulties with diet adherence	[172]
								TC −11			
									FPG −5		
							DBP −8	LDL-C −5			
									HOMA-IR −0.72		
								HDL-C 0			
				CR *n* = 34 75% of energy needs each day	−1.7	−1	SBP −1	TG −40	FI −1.56 μU/mL	No complaint due to difficulties with diet adherence	
								TC −8			
									FPG 0		
							DBP −5	LDL-C −1			
									HOMA-IR −0.39		
								HDL-C 0			
	Randomized controlled trial NCT03608800	39 subjects with MetS, 21 males and 18 females aged 30–50 y	8	ADF *n* = 21 75% energy restriction for 2 non-consecutive days a week and an ad libitum diet the other 5 days	−3.5	−2.5	SBP −5.3	TG –0.22	FI NA	91.3% of participants were compliant	[173]
								TC –0.04			
									FGP NA		
							DBP −2.5	LDL-C +0.02			
									HOMA-IR −0.75		
								HDL-C +0.5			
				CONTROL *n* = 18 routine diet without dietary instructions	−1.2	−1.1	SBP −4.9	TG 0	FI NA	78.3% of participants were compliant	
								TC −0.27			
									FGP NA		
							DBP −2.1	LDL-C +0.55			
									HOMA-IR −0.09		
								HDL-C +0.16			

DBP = diastolic blood pressure; SBP = systolic blood pressure; FI = fasting insulin; FPG = fasting plasma glucose; HDL-C = high-density lipoprotein cholesterol; HOMA-IR = homeostasis model assessment for insulin resistance; LDL-C = low-density lipoprotein cholesterol; NA = not applicable (parameter not measured); TC = total cholesterol; TG = triglycerides; WC = waist circumference.

## Data Availability

No new data were created or analyzed in this study. Data sharing is not applicable to this article.
